# Balanced polymorphism in a floral transcription factor underlies the ancient rhythm of daily sex alternation in avocado

**DOI:** 10.1073/pnas.2606876123

**Published:** 2026-07-28

**Authors:** Jeffrey S. Groh, Marllon F. Soares dos Santos, Emmanuel Avila de Dios, Gracie Ackerman, Edwin Solares, Rodrigo A. Iturrieta, Eric Focht, Danelle Seymour, Brandon S. Gaut, Mary Lu Arpaia, Graham Coop

**Affiliations:** ^a^ Department of Evolution and Ecology, University of California, Davis, CA 95616; ^b^ Center for Population Biology, University of California, Davis, CA 95616; ^c^ Miller Institute for Basic Research in Science, University of California, Berkeley, CA 94720; ^d^ Botany and Plant Sciences Department, University of California, Riverside, CA 92521; ^e^ Department of Computer Sciences and Engineering, University of California, San Diego, CA 92093; ^f^ Department of Ecology and Evolutionary Biology, University of California, Irvine, CA 92697

**Keywords:** avocado, heterodichogamy, R2R3 MYB, flowering time, mating system

## Abstract

In avocado and certain wild relatives in Lauraceae, a highly synchronized daily rhythm of floral sex timing promotes cross-pollination between two hermaphroditic flowering types. A-type plants present female-phase flowers in the morning and male-phase flowers in the afternoon, while B-types show the complementary pattern—a form of heterodichogamy. We mapped this dimorphism to a pair of dominant and recessive haplotypes at *SDMYB*, which belongs to a subgroup of R2R3 MYB transcription factors established as key regulators of floral maturation with links to circadian hormone signaling. Rhythmic diel *SDMYB* expression is associated with biphasic floral anthesis, and the dominant allele, which contains nonsynonymous changes in conserved functional domains, exhibits a *cis*-regulated phase delay, corresponding to the delayed second anthesis of A-type flowers. The *SDMYB* haplotypes form an ancient trans-species polymorphism, maintained by negative frequency-dependent balancing selection over 42 My, and they segregate in at least 26 nonavocado species, including in a genus where this mating system has not been reported. Although exceptionally old, the polymorphism is absent in other magnoliids with highly similar mating systems, suggesting daily forms of heterodichogamy can convergently evolve when rhythmic floral movements are coupled with the temporal separation of sexes.

## Significance

Hermaphroditic plants commonly evolve mechanisms to resolve conflict between male and female components of fitness, for example through temporal separation of the sexes. In avocado, A-type and B-type varieties open male- and female-phase flowers in reverse order throughout the day, a system that has convergently evolved in several plant lineages and is maintained at equilibrium by negative frequency-dependent selection. We present evidence that these flowering types are transcriptionally regulated by divergent alleles of the floral transcription factor *SDMYB*. These alleles occur in dozens of wild relatives of avocado, defining a polymorphism under long-term balancing selection for over 40 My. These findings contribute to our understanding of mating type evolution in hermaphrodites and will facilitate future avocado breeding efforts.

*“The daily rhythmic alternation of sexes... for the entire plant and the reciprocation of these changes in certain groups of plants reach a perfection of physiological regulation in avocados that is unapproached, as far as is now known, in any other group of plants.”*—A.B. Stout, 1927

Flowers are a key innovation that have contributed to the rapid diversification and ecological dominance of angiosperms (1–3), and they play vital roles in agricultural systems and human cultures. Flowering time and maturation are tightly regulated by endogenous biological rhythms and coupled to seasonal and daily environmental cues to coordinate flowering with conditions that maximize reproductive success (4, 5). Considerable progress has been made in elaborating gene regulatory networks that govern the transition to flowering, flower organ development, and flower maturation in model systems (6–8), yet connecting these findings to the genetic targets of mating adaptations in natural and agricultural systems remains an open challenge.

A widespread feature of angiosperm flowering is dichogamy—the temporal separation of male and female sexual maturity within a hermaphrodite individual. The high prevalence of dichogamy may have several adaptive causes, but two prevailing hypotheses are the avoidance of pollen-pistil interference and avoidance of self-pollination (9, 10). Dichogamy is most often monomorphic within populations, with all individuals separating male and female in the same direction with sufficient spread among individuals to permit cross-pollination. However, in at least 14 angiosperm families, some lineages have further evolved dimorphism for the direction of dichogamy (11–13). Such dimorphism, known as heterodichogamy, facilitates the evolution of a balanced sex ratio (14), analogous to how Fisherian sex ratio selection maintains a 1:1 sex ratio in many species. Heterodichogamy polymorphisms are part of a broader class of mating-type polymorphisms where disassortative mating generates rare-mating-type advantage, a form of negative frequency-dependent balancing selection capable of maintaining genetic and phenotypic variation over deep evolutionary time scales. Research on reproductive polymorphisms associated with dioecy, heterostyly, and self-incompatibility has contributed fundamental insights into the fitness advantages of outcrossing, the role of ecology in shaping mating strategies, the molecular basis of self-recognition, and the formation of supergenes (15–20).

Heterodichogamy has evolved on both seasonal and daily timescales (12). Recent work has begun to uncover the genetic basis of both seasonal and daily heterodichogamy, revealing a surprising diversity of genetic mechanisms that govern its inheritance. Seasonal heterodichogamy is best known in Juglandaceae. At least four different genetic systems were recently shown to underlie the same phenotypic polymorphism present throughout most of the family (21, 22, 23), suggesting the possibility of turnover from an ancestral genetic system. The functional changes involve genes linked to carbohydrate signaling and meristem regulation, and operate early in flower development, seemingly via small RNAs in at least two genera. The genetic basis of one form of daily heterodichogamy, flexistyly, was recently characterized in Zingiberaceae (24). In this system, two morphs show synchronized patterns of style movement and anther dehiscence, controlled by rhythmic expression of a single gene with pleiotropic effects. Although seasonal and daily heterodichogamy operate on different developmental timescales, both are governed by Mendelian inheritance of oligogenic regions, suggesting repeatable genomic outcomes in the evolution of heterodichogamy from dichogamy. Aside from these few examples, however, the regulatory basis of heterodichogamy remains poorly known, and may reveal genetic constraints to sex ratio selection in dichogamous populations.

**Fig. 1.**
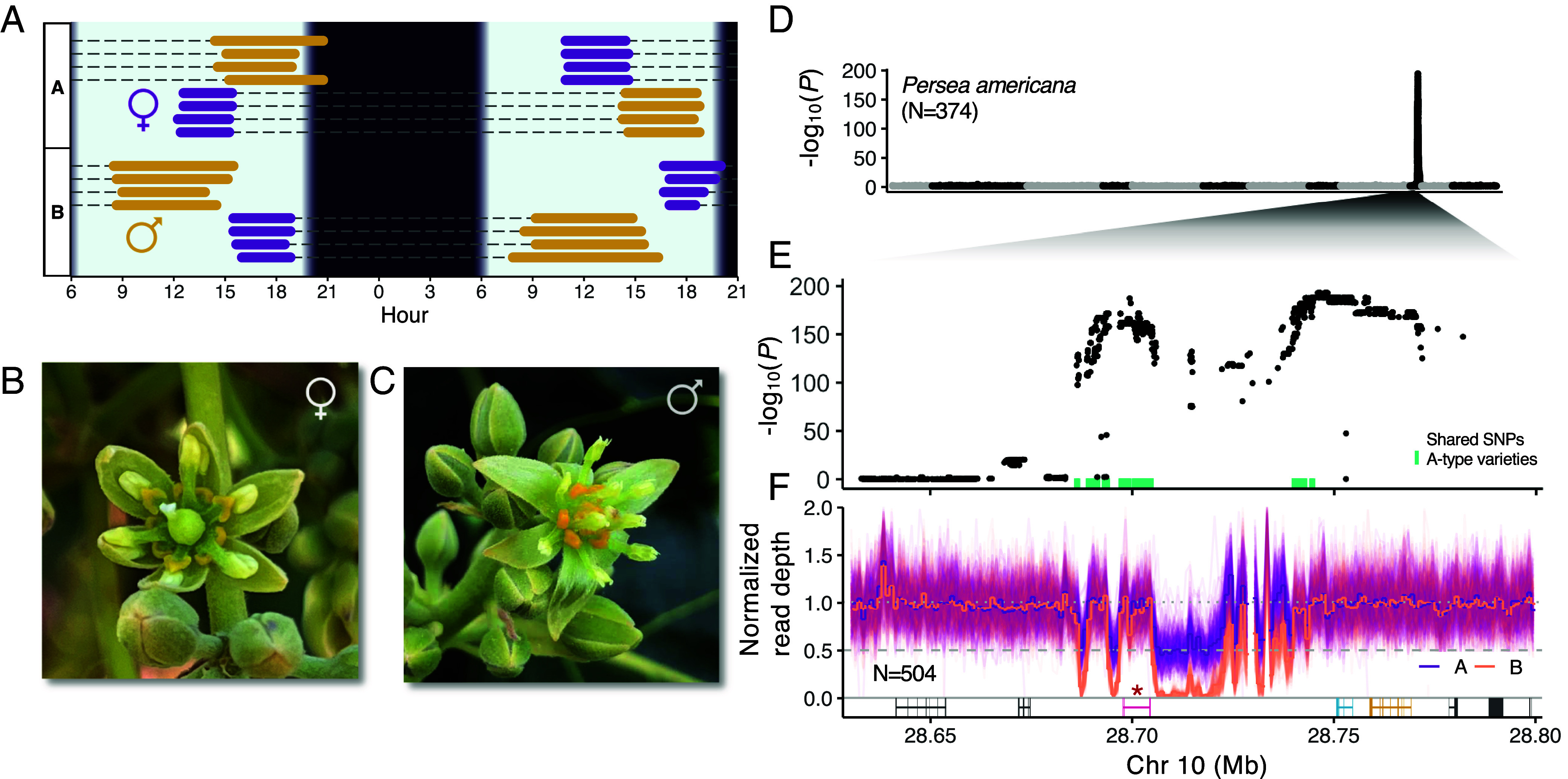
A single-gene Mendelian locus controls synchronized dichogamy in avocado (*Persea americana*). (*A*) Diagram of flowering phases for one A-type avocado variety (*Top*) and one B-type variety (*Bottom*) over two consecutive days at UC Lindcove Research and Extension Center, Exeter, CA on April 10–11, 2024. Colored horizontal bars indicate periods of anthesis of individual flowers (purple—female phase, gold—male phase), and dotted gray lines represent when the same flowers are closed in between successive openings. Note anther dehiscence occurs 1 to 2 h after the onset of second anthesis. (*B* and *C*) Flowers of *P. americana* in female (*B*) and male phase (*C*). (*D*) Association mapping for dichogamy type in an A × B cross. Alternating black and gray points represent SNPs on different chromosomes. (*E*) Close-up view of the association peak. Colored tick marks at bottom indicate the locations of shared SNP polymorphisms among A-type heterozygotes from an independent dataset. (*F*) Normalized read depth in 1 kb windows. Lines for each individual are partially transparent, solid lines show averages for each type. Gene models shown at bottom, *SDMYB* marked by asterisk. Photos in (*B*) and (*C*) by J.S.G. and M.F.S.d.S., respectively.

The avocado (*Persea americana*) exhibits a remarkable example of heterodichogamy in its daily flowering rhythm (Fig. 1*A*). On a given tree, two sets of flowers will open (anthesis) and close synchronously at different times during a single day (Movies S1–S3). These two sets of flowers differ in their functional sex—one receiving pollen and the other releasing pollen. Thus, a hermaphroditic tree will be functionally male or female at different times of day. Evidence of inbreeding depression in selfed avocado fruits (25) suggests the avoidance of self-pollination through dichogamy is likely adaptive. Avocado plants are further classified into two types, A and B, corresponding to whether the first set of flowers to undergo anthesis on a given day is functionally female (A-type) or male (B-type) (26). Thus under typical conditions, A-type avocado varieties are functionally female in the morning and functionally male in the afternoon, while B-type varieties show the complementary pattern, promoting crossing between A- and B-type varieties.

This reciprocal rhythm of alternating sexual function at the level of individual plants results from temporal offsets in a biphasic rhythm of individual flowers. Each flower is cosexual and opens twice over two consecutive days. The flower first opens in female phase, closes overnight, and reopens the next day in male phase. In A-type plants, the first anthesis occurs earlier on the first day, and the second anthesis occurs later on the second day, as compared to B-types (Fig. 1*A*). A large avocado tree may possess in excess of a million flowers, and the opening of flowers is staggered over successive days throughout the flowering season. This leads to a regular daily rhythm of alternating sexes in reversed directions between A- and B-types.

Since the original description of the avocado mating system (26), highly similar mating systems have been reported in several members of the laurel family (Lauraceae), including other members of tribe Perseeae (27–29, Movies S4–S7), as well as in more distant relatives of other tribes including true cinnamon (30, 31). These observations led some authors to suggest that this heterodichogamous mating system—termed synchronized dichogamy—might be ancestral to Lauraceae (30), a large family originating in the Cretaceous whose ca. 3,000 modern representatives are almost all long-lived woody perennials distributed throughout the tropics and subtropics (32). On the other hand, well-documented reports of synchronized dichogamy in Lauraceae remain sparse, and the occurrence of analogous types of heterodichogamy in other magnoliid families (12, 33, 34) suggests that this mating system might have instead repeatedly evolved within Lauraceae.

The avocado mating system has been both a point of fascination to botanists, as well as a challenge for growers, as both A- and B-type varieties are often planted together to maximize yield. The regularity and timing of the two phases is known to depend on both the diel light–dark cycle and temperature, contributing to variable crop yields of A- and B-types in different climates (35–38). Elucidation of the genetic regulation of synchronized dichogamy in avocado could thus facilitate enhanced breeding efforts for a globally significant crop at the center of a multibillion dollar industry. Previous work established that inheritance of dichogamy type in avocado is governed by a single locus with a dominant allele for A-type flowering (39). Here, we utilize a large mapping population of 374 resequenced avocado individuals together with de novo genome assemblies and time courses of gene expression from several species to identify the genomic and regulatory basis of synchronized dichogamy in avocado and its wild relatives. We find that synchronized dichogamy in avocado is linked to differences in rhythmic diel expression of alleles at a single flower-specific R2R3 MYB transcription factor, and that these alleles are both ancient and widespread throughout many species of Perseeae.

## Results

### Mapping the Synchronized Dichogamy Locus in Avocado.

We performed a genome-wide association study (GWAS) for A vs. B flowering type in 374 resequenced and imputed progeny from a reciprocal, open-pollinated cross (*Materials and Methods*) between the A-type heterozygote variety “Gem®” (PN 3-29-5) and the B-type variety “Luna UCR®” (PN BL516). This analysis revealed a single strong association peak on chromosome 10 (Fig. 1 *D* and *E*) close to a known broad QTL peak for flowering type (39). For convenience, we refer to this association region as the SD-locus (for Synchronized Dichogamy), and to the dominant and recessive alleles as *A1* and *A2*, respectively. In these and additional newly phenotyped and resequenced maternal sibs (N = 504 total), A- and B-types show clear differences in average read depth against a haplotype-resolved assembly of *P. americana* “Hass” (40), an A-type heterozygote (Fig. 1*F*). These patterns indicated the presence of structural variation at the SD-locus and confirmed the dominance of the *A1* allele found in A-type plants.

As the mapping population progeny originated from open pollination of the maternal trees by insects, we used offspring genotype ratios to estimate the strength of disassortative mating (matings between A- and B-types rather than within types). Contrary to the expectation under equal mating probabilities between vs. within types, we found a deficit of *A1/A1* homozygotes among progeny of the heterozygote A-type maternal variety ($P=2.0\;\times \;10^{-4}$, $\chi^{2}=17.04$, d.f. = 2) and a deficit of *A2/A2* homozygotes among progeny of the B-type maternal variety ($P=1.4\;\times \;10^{-14}$, $\chi^{2}=59.18$, d.f. = 1). Using these offspring genotype ratios, we estimate 85% and 88% of pollen parents of the progeny of A- and B-type maternal trees, respectively, were of the opposite flowering type to the maternal tree. This result supports the notion that dichogamy and reciprocal sex expression of A- and B-types promotes disassortative mating. We expect such a mechanism to generate rare-mating-type advantage, which could underlie the long-term maintenance of the synchronized dichogamy polymorphism in natural populations.

Three genes within the association peak were considered initial candidates (Fig. 1*F*, bottom in color). However, using published resequencing data from additional phenotyped avocado varieties (41, 42), we found that the region of shared heterozygosity among a broader diversity of A-type varieties narrows the boundary of the SD-locus to a region overlapping only one of these genes (Fig. 1*E*, *Bottom*). Conserved domains identified this gene as a member of the flower-specific SG19 subfamily of R2R3 MYB transcription factors (*SI Appendix*, Fig. S2 *A* and *D*), which have been extensively characterized in eudicot and monocot model systems and shown to act as key regulators of floral opening, sex organ maturation, and the production of nectar, pigments, and volatiles (e.g., refs. 6 and 43, 44, 45, 46, 47, 48, 49, 50, 51 and references within). Notably, this candidate gene is the closest avocado homolog of the *OsMYB8* gene in rice, where natural variation in the promoter region controls a difference in diurnal floret opening time between *indica* and *japonica* ecotypes (51). The avocado gene shows flower-specific expression, and is highly expressed in flowers (99.86th percentile among genes expressed in flowers), in clear contrast to the other two genes near the association peak (*SI Appendix*, Fig. S2 *E* and *F*). While the present manuscript was publicly available as a preprint and under editorial consideration, the same gene region was independently mapped in a separate panel of avocado varieties (52), providing independent evidence of its association with flowering type. As variation at this gene is the strongest candidate for regulating synchronized dichogamy in avocado, we refer to it as *SYNCHRONIZED DICHOGAMY MYB* (*SDMYB*).

While the molecular function of SG19 R2R3 MYB genes has not been previously investigated in any magnoliid to our knowledge, studies in eudicot and monocot systems have demonstrated that they localize to the nucleus and function as transcription factors (e.g., refs. 48, 51, 53, and 54). Both the R2R3 DNA binding domain and the transcriptional activation domain (TAD) are required for transcriptional activation of target genes (reviewed in ref. 50). Consistent with this putative function, both avocado *SDMYB* alleles contain conserved R2R3 DNA binding domains and characteristic SG19 TADs with a predicted alpha-helix (44) (*SI Appendix*, Fig. S2 *A*–*C*). The two avocado protein alleles have highly similar predicted 3D structures (*SI Appendix*, Fig. S2 *B* and *C*), yet notably differ by an aspartate to glycine substitution at a strongly conserved site within the R2R3 DNA binding domain of the dominant allele, as well as a frame-shift mutation within the TAD of the dominant allele, suggesting possible functional changes. Given the distinct temporal flowering rhythms of A- and B-types, we hypothesized that these two *SDMYB* alleles should show distinct diel expression patterns.

### Temporal Regulation of SDMYB Underlies Phase Synchrony of A and B Flowering Types.

**Fig. 2.**
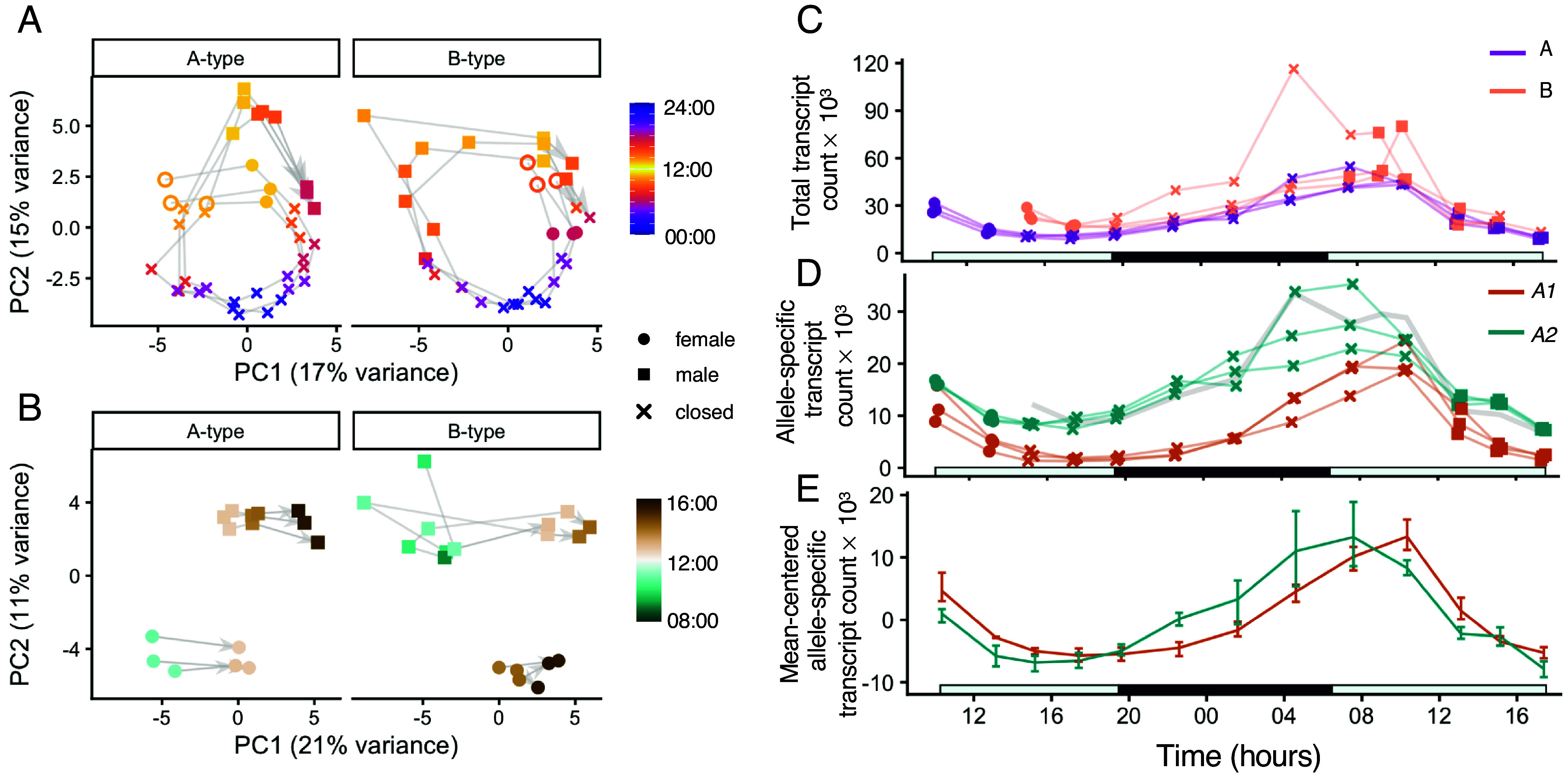
Gene expression rhythms in avocado flowers and allele-specific regulation of *SDMYB*. (*A*) Principal components analysis (PCA) of genome-wide transcript counts. Each point represents an individual flower colored by the time of day of sampling, shape indicates developmental phase. Lines connect samples from the same individual, progressing along the developmental time course of flower maturation from open circle to arrowhead. (*B*) PCA of transcript counts for open flowers only. (*C*) Total expression of *SDMYB* transcripts (both alleles) following onset of first anthesis. Lines connect expression data from flowers sampled at different time points from the same individual. Point shapes indicate developmental phase of each sampled flower as in (*A*). (*D*) Allele-specific expression of *SDMYB* alleles in heterozygote A-type individuals of avocado, the same individuals shown in purple in (*C*) with each individual having two lines to represent both alleles. The gray line shows half of the average expression of *SDMYB* in B-type individuals from (*C*). (*E*) Average of mean-centered allele trajectories of heterozygotes from (*D*) reveals a phase delay of the *A1* allele.

To investigate the regulatory basis of synchronized dichogamy in avocado, we constructed a time course of gene expression across the period of floral maturation beginning from the onset of first anthesis to the end of second anthesis (Fig. 1*A*). To do this, we sampled flowers across developmental stages from three biological replicates of each morph every 3 h spanning a 24 h interval (*SI Appendix*, Tables S1 and S2). Using genome-wide transcript counts, we found all individuals show a clock-like oscillation along the first two principal components according to the time of day of sampling (Fig. 2*A*). Principal Component 1 (PC1, 17% of variance) captures expression variation associated with morning vs. afternoon while PC2 (15% of variance) captures variation associated with night vs. day. Thus, despite the conspicuous offset timing of sex expression between A- and B-types, the major axes of genome-wide expression variance in flowers reveal a largely shared diel trajectory. Consistent with this, we find that 50% of all flower-expressed genes (24% of all annotated nuclear genes) showed significant rhythmicity in both A- and B-types (FDR = 0.05). Subsetting the data to only open flowers, PC2 (11% variance) separates samples in male vs. female phase (Fig. 2*B*), suggesting that the core transcriptional program regulating within-flower dichogamy is largely shared between types.

We found that *SDMYB* is differentially expressed between types across the time course (P=0.039) and exhibits striking temporal variation in allele-specific expression (Fig. 2 *C* and *D*), in contrast to the other genes near the SD-locus (*SI Appendix*, Fig. S2 *G* and *H*), further supporting our identification of *SDMYB* as the best functional candidate. In both avocado types, *SDMYB* expression was detected as significantly rhythmic over the 24 h cycle ($P=2.73\;\times \;10^{-8}$), with our sampling capturing a clear peak and trough in expression. Expression declines immediately following the onset of first anthesis, then beginning in the afternoon gradually increases toward a local maximum just prior to or coincident with second anthesis on the following day (Fig. 2*C*). Although we could not determine the expression level of *SDMYB* in flowers prior to their first anthesis (the first sampling point corresponds to the start of first anthesis), our data appear consistent with a model whereby *SDMYB* expression must reach a threshold level for the flower to open, mirroring a pattern seen for orthologs of *SDMYB* in *Petunia* and *Nicotiana* (45, 53).

Total *SDMYB* expression is on average lower in A-types compared to B-types (Fig. 2*C*, P=0.035, $\chi^{2}=4.28$, d.f. = 1). This difference is accounted for by lower expression of the *A1* allele in heterozygotes (Fig. 2*D*, $P<2.2\;\times \;10^{16}$, $\chi^{2}=141.8$, d.f. = 1), whereas the *A2* allele in heterozygotes shows expression levels largely consistent with the haploid equivalent in *A2/A2* B-types (Fig. 2*D*, gray line). Because the genomic context of these alleles is randomized outside of the SD-locus haplotypes (Fig. 1*D*), these allele-specific expression patterns imply there are *cis*-acting regulatory differences associated with the haplotypes. Expression of the *A2* allele in heterozygotes did not significantly differ from the haploid equivalent in *A2/A2* B-types in overall expression level, amplitude, nor phase; however, when testing each timepoint individually, we noted a significant difference in expression between *A2* in A-types and the haploid equivalent in B-types at only the first time point (P=0.018, Bonferroni corrected), suggesting the regulation of *A2* could be subtly altered in a heterozygous background. Thus we do not exclude the possibility that SD-locus haplotypes affect *SDMYB* expression in *trans*. Most notably, we identified a phase shift between the two alleles, with the *A1* allele showing an average 2-h phase delay in heterozygotes (Fig. 2*E*, cosinor regression $P=1.4\;\times \;10^{-4}$). As this allele-specific expression delay closely corresponds to the delayed timing of second anthesis of A-types, the data strongly suggest that synchronized dichogamy in avocado is determined in part by *cis*-regulatory divergence between *SDMYB* alleles.

### The SD-Locus Is an Ancient Trans-Species Polymorphism.

Synchronized dichogamy has been reported in several members of Lauraceae, including in close relatives of avocado in *Persea* subg. *Eriodaphne* and *Machilus* (tribe Perseeae) (27, 28) and in more distant relatives in tribes Cinnamomeae and Mezilaureae (30, 31). To identify whether the avocado SD-locus might also regulate this mating system in other species, we first generated haplotype-resolved genome assemblies (*SI Appendix*, Table S3) from phenotyped individuals of *Machilus thunbergii* (A-type, Movie S4) and *P. caerulea* (A-type, Movie S6), as well as an individual of *P. podadenia* (unknown type), together with *P. americana* “Puebla,” another A-type avocado variety. We used these together with published Lauraceae genome assemblies and both published and new resequencing data (*SI Appendix*, Table S4) to better resolve relationships within Perseeae, and to provide a phylogenetic framework for probing the evolutionary history of the SD-locus polymorphism.

**Fig. 3.**
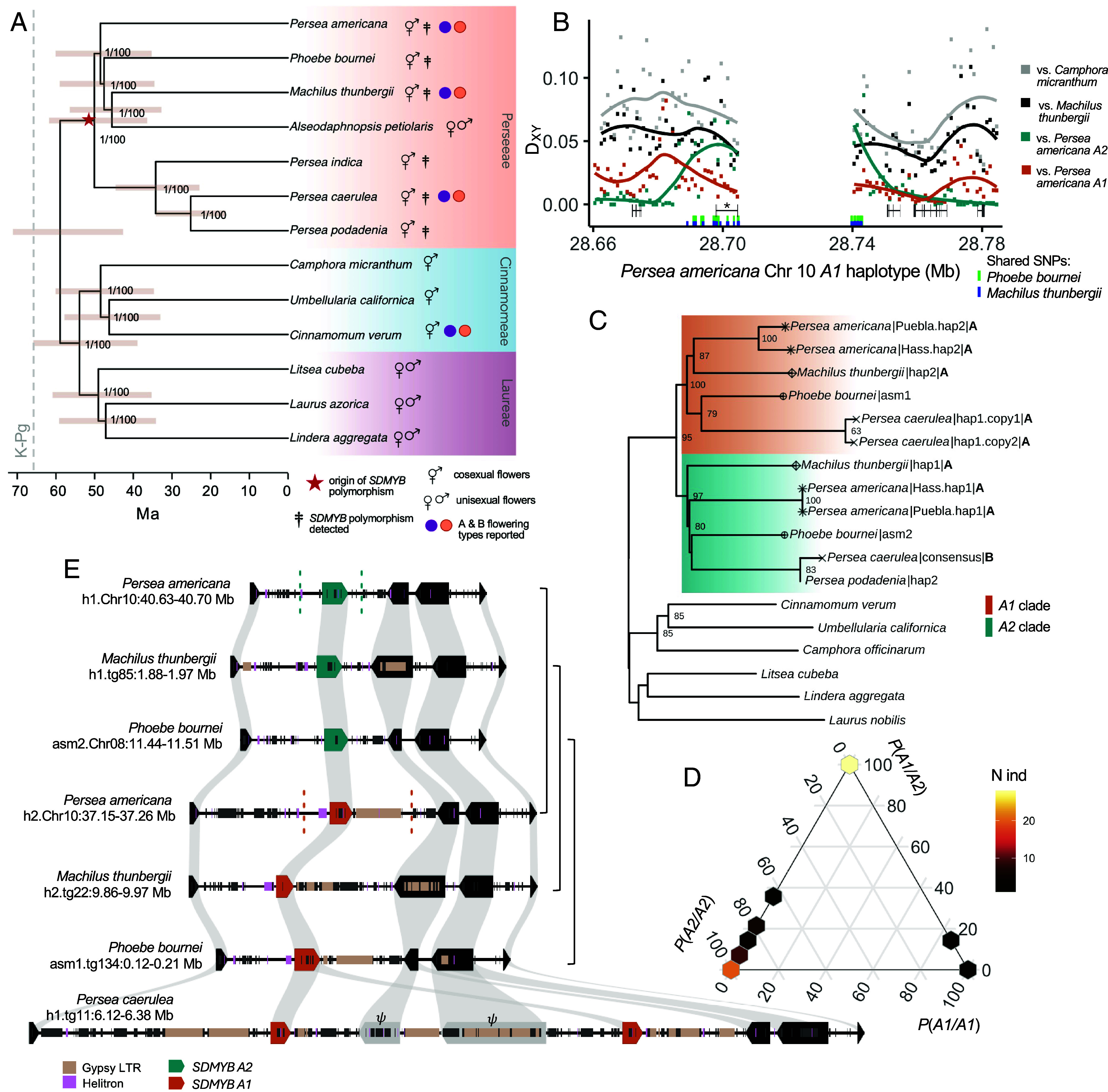
Trans-species polymorphism and structural variation of SD-locus haplotypes. (*A*) A phylogeny of three tribes of Lauraceae (see also *SI Appendix*, Fig. S3). Axis at bottom indicates timescale, and gray error bars represent 95% HPD intervals. Internal nodes are labeled with Local Posterior Probability support values/bootstrap support values. The red star indicates approximate divergence time of the *SDMYB* alleles. (*B*) Nucleotide divergence between the avocado Hass *A1* haplotype and other Lauraceae genomes. Each genome compared to Hass is represented using a different color. The orange line indicates comparison between the Hass and Puebla *A1* haplotypes. Squares indicate values measured in 1 kb windows, and lines show LOESS smoothed curves. Gene models are shown at bottom (*SDMYB* denoted with asterisk) and the locations of shared trans-species SNPs between avocado and *Phoebe bournei*/*M. thunbergii* are indicated in colored tick marks. (*C*) Maximum likelihood phylogeny of the SD-locus, rooted with *Sextonia rubra*. Node labels indicate bootstrap support for support values above 60%. Tip shapes highlight species segregating for the trans-species polymorphism. Where known, flowering types (A/B) are indicated after species name and assembly identifier. (*D*) Ternary plot showing genotype posterior probabilities for the SD-locus for 72 individuals representing 56 species of Perseeae. Hexagons indicate the locations of data points, with color indicating the number of individuals at a location. (*E*) Synteny plot of the local region around *SDMYB* for assemblies from several Perseeae species. Pointed rectangles indicate gene models and direction of transcription, with ribbons connecting orthologs. *SDMYB* is colored by allele (orange—*A1*, teal—*A2*). Narrow rectangles along haplotypes show repeat annotations, with two particular classes highlighted in color. Braces at right connect haplotypes from the same species. Dotted lines on *P. americana* haplotypes indicate the region of trans-species polymorphism surrounding *SDMYB*. ψ in the *P. caerulea* assembly denotes predicted pseudogenes.

Phylogenetic analysis using 7,842 single copy orthologs confirmed reciprocal monophyly of the three accepted Lauraceae tribes represented in our dataset (Fig. 3*A* and *SI Appendix*, Fig. S3). However, our phylogeny demonstrates unambiguously that *Persea* is not monophyletic, which has been previously recognized (55). While a taxonomic revision of *Persea* is not our focus, our analysis appears consistent with the suggestion that *Persea* subg. *Persea* and *Persea* subg. *Eriodaphne* should be treated as separate monophyletic genera (56).

We first examined the SD-locus in close relatives of avocado known to be phenotypically polymorphic for A- and B-types—*M. thunbergii* (28), and *P. caerulea* (27). Between the two assembled haplotypes of the A-type *M. thunbergii*, we observed a high concentration of shared heterozygous sites with A-type avocado varieties closely aligning with the boundaries of the avocado SD-locus (Fig. 3*B*, *Bottom*, blue). This pattern is consistent with trans-species (i.e., across-species) polymorphism, a classic signature of long-term balancing selection where the coalescent time of alleles is older than the divergence time of the species they segregate in. We next determined that our A-type *P. caerulea* assembly individual was largely homozygous for *A1* variants at these same sites, consistent with it having an *A1/A1* genotype. RNA-seq data from a separate A-type individual of this species showed both *A1* and *A2* variants at these sites, while data from a B-type individual contained only *A2* variants, confirming that the avocado SD-locus SNPs segregate in *P. caerulea* with the same phenotypic association. The nonsynonymous substitution within the R2R3 DNA binding domain as well as the frame-shift mutation within the TAD of *SDMYB* are conserved differences between the *A1* and *A2* haplotypes across these species (*SI Appendix*, Fig. S2*A*), suggesting these arose early in the formation of the SD-locus haplotypes.

The narrow region of phenotype-associated trans-species polymorphism suggested these haplotypes are maintained by long-term balancing selection. Nucleotide divergence between the avocado haplotypes sharply increases at the boundaries of the SD-locus, reaching a level comparable to the divergence of *P. americana* and *M. thunbergii* (Fig. 3*B*), suggesting the haplotypes could have diverged in the common ancestor of Perseeae. A maximum likelihood phylogeny of the SD-locus supports that the sets of *A1* and *A2* haplotypes from across Perseeae are reciprocally monophyletic (99 and 88% bootstrap support) (Fig. 3*C*). To calibrate the age of their divergence, we time calibrated our core Lauraceae phylogeny grafted into a magnoliid backbone tree (*SI Appendix*, Fig. S3), leveraging information from several fossil calibrations (*SI Appendix*, Tables S5 and S6). Doing so, we infer an Eocene origin of tribe Perseeae (50.1 Ma, 95% HPD [36.4, 61.8]). Using the divergence of Perseeae from the Cinnamomeae-Laureae clade to calibrate the divergence of the SD-locus haplotypes, we obtain an estimate of 42.4 Ma, 95% HPD [30.7, 51.2]. This estimate is consistent with these haplotypes originating in the common ancestor of Perseeae, or possibly introgression in the very early history of this group.

Long-term maintenance of the SD-locus haplotypes by balancing selection since the common ancestor of Perseeae predicts that they should segregate in many of the ca. 400 species of Perseeae distributed throughout the tropics and subtropics. To test this hypothesis, we inferred SD-locus genotypes for a set of 72 individuals representing 56 species in Perseeae using published and newly generated sequence data (*SI Appendix*, Table S7) and diagnostic SD-locus polymorphisms within *SDMYB* exons. Doing so, we identified SD-locus haplotypes segregating in 26 non avocado species (Fig. 3*D* and *SI Appendix*, Table S7). The signal of shared exonic polymorphisms we detect is restricted to *SDMYB* and not seen in surrounding genes (*SI Appendix*, Fig. S5). In this cross species sample, *A1/A2* and *A2/A2* genotypes are both common (53% and 43%, respectively) whereas *A1/A1* homozygotes are rare (4%), consistent with the prediction that the SD-locus polymorphism is selectively maintained through disassortative mating in most of these species. Notably, we also identified *A1* and *A2* haplotypes contained within two independent genome assemblies from *Phoebe bournei* (57, 58) (Fig. 3*E*), and confirmed that the individual from which the *A1* haplotype assembly derived was heterozygous across most of the trans-species polymorphic SNPs identified at the SD-locus (Fig. 3*B*, *Bottom*, green).

While synchronized dichogamy is well-documented in true cinnamon (*Cinnamomum verum*) of tribe Cinnamomeae (31) and has also been reported in tribe Mezilaureae (30), our phylogeny inference (Fig. 3*C*) and divergence across the region (Fig. 3*B*) indicates that the SD-locus haplotypes are not as old as the divergence of Perseeae from other Lauraceae tribes. Further supporting this, we did not see the diagnostic *SDMYB* SNPs segregating in resequencing data from three individuals of true cinnamon, nor in resequencing data from 24 additional species in tribe Cinnamomeae (59). Finally, we examined a natural population of *Umbellularia californica* (California bay laurel) and found that it does not exhibit synchronized dichogamy (*SI Appendix*, Fig. S6). Altogether, these results indicate that the SD-locus polymorphism is not found within tribe Cinnamomeae and that synchronized dichogamy is not ubiquitous in this group, suggesting there could have been multiple independent origins of synchronized dichogamy within Lauraceae.

Haplotype-resolved genome assemblies revealed that structural differences between the *A1* and *A2* haplotypes are a shared feature across Perseeae species (Fig. 3*E* and *SI Appendix*, Fig. S4 *A*–*C*). Dominant *A1* haplotypes show a moderate increase in length compared to *A2*, accounted for by transposable elements—most notably Gypsy-LTR-derived sequence downstream of *SDMYB*. In the *A1/A1P. caerulea* homozygote, both haplotypes contain a tandem duplication of the *A1* allele of *SDMYB*, which we did not detect in RNAseq data from a B-type *P. caerulea* individual nor in the *A2* long-read haplotypes from our *P. podadenia* assembly individual, a close relative. While these *SDMYB* paralogs show identical coding sequence, two other genes in the duplicated segment appear pseudogenized (*SI Appendix*, Fig. S4*E*), suggesting that both *SDMYB* paralogs are selectively maintained. Despite the observed structural differences between haplotypes, it is noteworthy that the dichogamy-linked region has not expanded into a large heteromorphic region, mirroring an emerging trend identified in other heterodichogamy systems (21–24) and many plant sex determining regions (e.g., 60, reviewed in ref. 61).

**Fig. 4.**
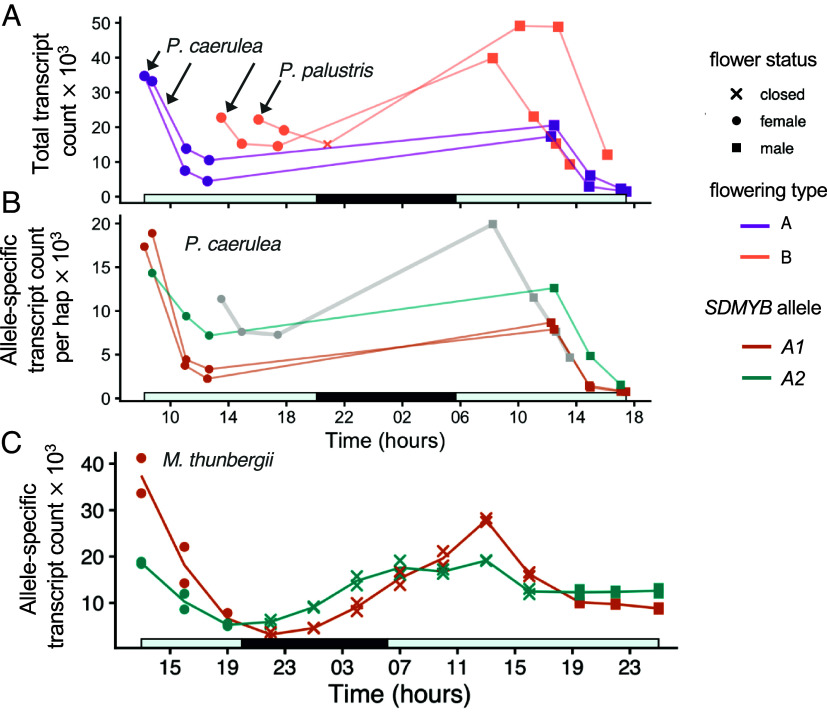
Conserved allele-specific regulation of *SDMYB* across Perseeae. (*A*) Time course of total *SDMYB* transcription (both alleles) for three individuals of *Persea caerulea* and one individual of *P. palustris*. The A-type *P. caerulea* individual showing lower average expression of *SDMYB* (leftmost gray arrow) is an *A1/A1* homozygote while the other A-type individual of this species is an *A1/A2* heterozygote. (*B*) Time course of allele-specific *SDMYB* expression for the three *P. caerulea* individuals from (*A*). Transcript counts are normalized to haplotype dosage to facilitate comparison of *A1* expression between the homozygote and heterozygote. The gray line shows one half the expression of the *A2* allele seen in the B-type individual from (*A*). (*C*) Time course of *SDMYB* allele-specific expression for a single *M. thunbergii* A-type heterozygote. Each point represents an expression measurement from a separate flower, and lines show average of replicate flowers.

To test whether *A1* and *A2* alleles of *SDMYB* are differentially regulated in additional species, we generated additional time courses of floral gene expression from *Persea* subg. *Eriodaphne* and *M. thunbergii* (Fig. 4). Overall, these data support that *SDMYB* expression peaks in correspondence with anthesis, and that the *A1* and *A2* alleles have distinct diel expression patterns. In *Persea* subg. *Eriodaphne*, data from two A-type *P. caerulea* (one homozygote and one heterozygote), a B-type of *P. caerulea* and a B-type of *P. palustris* support lower overall expression of the *A1* allele as was seen in avocado. Our courser sampling in this group lacks power to detect a phase difference between alleles, but the data are nonetheless consistent with this possibility. We sampled flowers of an A-type *M. thunbergii* at higher temporal density and recovered a phase shift similar to that seen in avocado—in this cae, a 2.57-h phase delay in the *A1* allele (*P* = $6.49\times 10^{-4}$). Here, the *A1* allele does not exhibit lower average expression, but it does show significantly larger amplitude (*P* = $5.97\times 10^{-3}$). Taken together, these results indicate that the association between SD-locus haplotypes and allele-specific expression of *SDMYB* is shared among avocado and its wild relatives, suggesting that the mechanistic basis of synchronized dichogamy is evolutionarily conserved.

## Discussion

We have shown that synchronized dichogamy in avocado and its wild relatives is linked to diel expression rhythms of two ancient alleles of the floral transcription factor *SDMYB*. Our findings provide a genetic explanation for a century-old mystery of avocado pollination, anticipated by Stout (1927) who predicted that *“such decided and specific differences in action can only be due to differences in the inherent constitution of the two groups of varieties.”* Several aspects of the regulatory mechanism nonetheless remain to be solved.

First, it seems likely that the conserved non-coding polymorphisms within the SD-locus haplotypes mediate allele-specific expression of *SDMYB*. How do the offset rhythms of *SDMYB* alleles result from differential responses to shared upstream regulators? As the avocado flowering system appears at least partly linked to circadian regulation (36), and other SG19 MYBs are transcriptionally regulated by clock-regulated jasmonate signaling components (6, 49, 62–64), similar signaling components may be involved here. Separately, morph-specific disruptions to the avocado flowering rhythm by light and temperature (36, 65) suggest the offset rhythms of *SDMYB* alleles may be in part contingent on environmental cues. Consistent with this idea, expression of some SG19 MYBs has been found to be light- or heat-sensitive (54, 66). The expression differences between these alleles could also result in part from transcriptional regulation of one allele by the other, either directly or indirectly.

Second, while the delayed rhythm of the *A1* allele may explain the delay of second anthesis of A-types, it is not sufficient to explain the earlier first anthesis of A-types. Thus, we speculate that the bidirectional offsets of sex phases between flowering types derives from a combination of the differential *SDMYB* rhythmicity following first anthesis, as shown here, together with allele-mediated effects on development in response to an additional time-varying factor. Because our developmental time course does not include flower buds prior to their first anthesis, we cannot exclude the possibility that the observed phase shift is specific to the period following the onset of first anthesis.

Expression differences alone are unlikely to be the sole basis of synchronized dichogamy in these species. The nonsynonymous trans-species polymorphisms in otherwise highly conserved domains of SDMYB suggest that the protein alleles may have partially diverged in function. These amino acid differences might modulate a differential response to fluctuating concentrations of hormone signaling components or transcription-activation binding partners (46, 47, 67, 68). Given that the expression of *A2* is not markedly changed in the heterozygote background, particularly across second anthesis (Fig. 2*D*), differential protein–protein interactions could potentially form the mechanistic basis of dominance of the *A1* allele. One candidate set of binding partners are the JAZ proteins, jasmonate-responsive negative regulators that directly interact with SG19 MYBs to regulate flower maturation in Arabidopsis (46). Interestingly, some avocado homologs of the *Arabidopsis* JAZ proteins exhibit divergent expression rhythms between A- and B-types, implying these are regulated by *SDMYB* (*SI Appendix*, Fig. S7*A*). Because each flower must open and close twice, some such negative feedback loop could be a core component to the regulation of this mating system and biphasic flowering rhythms more generally.

Disassortative mating generated by heterodichogamy is hypothesized to drive strong negative frequency-dependent selection pushing populations toward a 1:1 morph ratio (14). The few natural samples of Perseeae examined to date suggest the two morphs occur in roughly equal proportions (27–29) (see also Fig. 3*D*), consistent with this prediction. Our identification of diagnostic trans-specific SNPs at the SD-locus now enables the estimation of morph frequencies and the strength of disassortative mating in a broader phylogenetic context. As synchronized dichogamy appears highly environmentally sensitive in avocado (36, 38, 65), it will be interesting to measure variability in the strength of disassortative mating across the Perseeae tribe, and explore whether buffering mechanisms have evolved in species that naturally experience a high degree of environmental variation.

Our comparative analyses of the Perseeae SD-locus further establish heterodichogamy as a powerful agent of long-term balancing selection. Clear examples of long-term balancing selection remain relatively rare in plants, and are most often associated with mating type loci and immune genes (69, 70). The SD-locus alleles likely diverged in the ancestor of Perseeae over 42 Mya, and appear to be common among the hundreds of species in Perseeae (Fig. 3*C*). The age of this system is comparable to the ages of the several genetic systems for heterodichogamy in Juglandaceae (independently balanced for >40 My, 22, 21). It is noteworthy that these estimates are on par with the oldest reported angiosperm sex chromosomes (e.g., refs. 71 and 72, reviewed in ref. 61), despite dioecy having evolved hundreds or thousands of times, whereas heterodichogamy is considerably more rare. Dioecy can be susceptible to rapid reversion to hermaphroditism under mate limitation (73). By contrast, the hermaphroditic condition of heterodichogamous plants may afford greater reproductive assurance and long-term stability of the causal polymorphisms. On the other hand, one way heterodichogamy might be lost is through disruptive selection on sex allocation and the evolution of dioecy (74, 75). The presence of unisexual flowers in *Alseodaphnopsis* (76), which our analysis shows is nested within Perseeae (Fig. 3*A*), suggests a promising system for investigating this evolutionary pathway to dioecy.

Although a highly similar form of synchronized dichogamy is found in tribe Cinnamomeae (31), our analyses indicate the mating system in cinnamon is not controlled by the same set of *SDMYB* alleles. In conjunction with the recent identification of the genetic control of flexistyly in tropical gingers (24), our results indicate that there are multiple developmental and gene regulatory avenues by which daily forms of heterodichogamy can evolve. As protogynous dichogamy is ubiquitous in magnoliids with cosexual flowers, and 2-day flowering rhythms linked to the day-night cycle are common (77), magnoliids may be predisposed to evolve daily forms of heterodichogamy, known in at least four families of this group (12, 33). We suggest that unlike seasonal forms of heterodichogamy, evolution of daily heterodichogamy is more likely to involve genes with late-acting roles in flower maturation. As *SDMYB* homologs are key regulators of diverse aspects of late-stage floral maturation (6, 50), we speculate that convergent evolution of daily heterodichogamy may often recruit genes closely interacting with *SDMYB*, or perhaps even homologs of *SDMYB* itself. Interestingly, the closest avocado homolog of *SMPED1*, a gene controlling flexistyly in *Alpinia mutica* (Zingiberaceae), shows differential regulation between flowering types at second anthesis (*SI Appendix*, Fig. S7*B*), consistent with this idea and its proposed role in anther dehiscence (24). As these pathways become increasingly well characterized, this knowledge will enable us to further define the genetic factors that promote or constrain the evolution of diverse mating strategies, and allow for genetic manipulation of key flowering traits in important agricultural crops.

Finally, the identification of the genetic basis underlying A- and B-type flowering behavior has direct implications for avocado breeding. Due to the protracted juvenile phase of avocado (5 to 12 y, ref. 78), breeding programs face long delays evaluating segregating populations and selecting parental lines. The genetic markers identified here will enable rapid screening for flowering type in seedlings, substantially reducing time, labor, and costs associated with field evaluation. Our findings further suggest *SDMYB* as a key target for directed breeding efforts, for instance to develop new pollen donors (pollenizers) for increased fruit set of Hass, the most widely planted and consumed avocado cultivar worldwide. Further genetic dissection of the avocado flowering mechanism will enhance strategies for breeding and orchard design, improving yields and sustainability of a globally significant crop.

## Materials and Methods

### Phenotyping.

An open-pollinated isolation block was planted with three trees each of Gem®(PN 3-29-5), an A-type heterozygote, and Luna UCR®(PN BL516), a B-type, at UC Riverside Agricultural Experiment Station, Riverside, CA in 1999. Seedlings were grafted onto clonal rootstock and planted in an experimental orchard at UC Riverside Agricultural Experiment Station in 2019–2022. During the first week of April 2024 (peak flowering in Riverside, CA) all 578 individuals in bloom were visited twice per day (9:00 AM to 12:00 PM and 2:00 PM to 5:00 PM) over four consecutive days to determine whether the tree expressed female or male function during each time period, and classify each tree as A or B flowering type. Phenotyping on each day was timed to avoid confounding effects of early morning cold-induced floral delay or incomplete anthesis.

### Association Mapping.

Sequencing reads of 712 avocado individuals were aligned to the *P. americana* Hass alternate assembly (GCA_031208655.1) (40) which we identified as containing the *A1* haplotype in the initial stages of our study. This included the two parents (mean depth 132×) and 710 progeny (7×). After variant calling, genotypes with GQ below 30 were set as missing. Prior to imputation, we removed samples with less than 2× mean depth, and plants with unintended parentage. Unintended parentage was determined by examining the proportion of variants per sample not found among the set of parental variants. We observed two largely discrete clusters suggesting a set of individuals had a paternal parent from outside the isolation block, and we set an ad hoc threshold of 0.0121 to remove these samples, leaving 486 offspring. Imputation was performed using AlphaPlantImpute2 (79). First, for each chromosome, a haplotype library for each chromosome was constructed from the genotypes of the two parents using AlphaPlantImpute2 -createlib. Offspring were then imputed using the founder haplotype library, setting -calling_threshold 0.9 to only retain high confidence imputed sites. Among the 486 imputed offspring, 374 were phenotyped for flowering type and were used in a GWAS. GWAS was performed in gemma 0.98.5 (80), removing sites with more than 5% missing data or less than 20% minor allele frequency. We reexamined published sequencing data from 23 avocado varieties (41, 42) and performed a separate GWAS using available phenotype information. This analysis identified the same locus with highly similar patterns of segregating read depth, confirming that the association identified in the mapping population was not specific to the mapping population family (*SI Appendix*, Fig. S1). To obtain functional annotations of candidate genes, we first used blastp and InterPro scan to identify genes with close homology. This identified *SDMYB* as a member of the SG19 subgroup of transcription factors (*SI Appendix*, Fig. S2 *A* and *D*).

### Analysis of Mating Patterns.

To estimate posterior genotype probabilities at the SD-locus for mapping population individuals, we used low coverage read counts at two diagnostic exonic SNPs within *SDMYB* approximately 6 kb apart. These SNPs are not spanned by single read pairs, so we used the sum of *A1* and *A2* reads over sites to compute a binomial genotype likelihood with a small probability of sequencing error ($1\;\times \;10^{-3}$). Genotype priors for each set of maternal sibs assumed pollen parents were equally likely to be A-type or B-type, and that *A1/A1* pollen donors were absent. A small number of Luna UCR®seedlings (<3%) showed zero *A2* reads over these SNPs, which we assumed to be a result of sampling error; these were called as heterozygotes. We ignored the set of trees from the first planting, as at least some of these were genotyped using SSRs and intentionally biased toward plants with the intended parentage. This left 244 and 307 Gem®and Luna UCR®seedlings, respectively. We used a Chi-Squared test for each set of seedlings with the same expected proportions as used in the genotype priors. (Note the expectation of zero *A1/A1* Luna UCR®seedlings removes one degree of freedom).

### Genome Assembly.

We generated de novo genome assemblies for *P. americana* Puebla from UC South Coast Research and Extension Center, *M. thunbergii* from Sonoma Botanical Garden, Glen Ellen, CA (acc. no. 1987.421A), *P. caerulea* from UC South Coast Research and Extension Center (plot 43, row 7, tree 1) and *Persea podadenia* from University of California Botanical Garden, Berkeley, CA (acc. no. 97.0505.363). High molecular weight DNA extraction, library preparation, and sequencing was performed by Signios Bio, Inc., Foster City, CA. We used hifiasm 0.20 (81) with default parameters to assemble CCS reads for each sample. For *P. americana*, we used ragtag (82) with default parameters to scaffold to an existing avocado assembly (83). Transposable elements were annotated using EDTA (84). Measures of assembly contiguity are given in *SI Appendix*, Table S3.

### Species Phylogeny Inference and Time Calibration.

Relationships within the ca. 400 species of Perseeae remain unresolved, and previous molecular phylogenetic studies have typically relied on single genes with limited fossil information for time calibration (55, 85–87). First, we identified a set of 7,862 single copy orthologs using fifteen genome assemblies spanning the core Lauraceae using OrthoFinder 2.5.5 (88), and recovered ortholog sequence from sixteen additional core Lauraceae species. We inferred gene trees from trimmed orthogroup alignments under a GTR+I+G4 substitution model using IQTree (89). We used both ASTRAL IV (90) and concatenation + maximum likelihood on the resulting gene trees, recovering identical topologies. We rooted the core-Lauraceae tree at the edge separating Perseeae from Laureae-Cinnamomeae and grafted this to a backbone tree inferred from a larger set of magnoliids. We time calibrated the tree using MCMCTree (91) using an alignment of 93 single-copy magnoliid orthologs and priors based on the fossil record of magnoliids (*SI Appendix*, Table S5). Additional details of phylogeny inference methods are given in *SI Appendix*, *Extended Methods*.

### Structure and Divergence of SD-Locus.

We computed read depth from alignment files for all phenotyped individuals (including those excluded from imputation or with unexpected parentage, N = 504) using samtools (92), averaged values in 1kb intervals, and normalized the windowed values by mean depth over Chr9. Synteny of surrounding genes in genome assemblies of avocado and relatives was determined using reciprocal blastn searches. We extracted the regions surrounding the SD-locus and generated dotplots from alignments using discontiguous megablast through NCBI. To measure nucleotide divergence of core Lauraceae genomes against the genome of *P. americana* Hass, we used AnchorWave (93) with settings -R 1 -Q 1 to align against the Hass alternate assembly (*A1* haplotype), and used a custom R script to calculate nucleotide divergence in 1 kb windows in coordinates of the reference. For visualization, we removed windows with fewer than 500 aligned base pairs in the reference and fit a LOESS smooth curve to the data with span = 0.75, separately on either side of the copy number variant. We also filtered the values immediately adjacent to the structural variant which we considered unrealistically high and likely a result of alignment error.

### Relationships Among SD-Locus Haplotypes.

To infer relationships within Lauraceae at the SD-locus, we first aligned i) approximately 6 kb upstream of the start codon of *SDMYB*, ii) the coding sequence of *SDMYB*, iii) intron 2 (approximately 6 kb), and a iv) ∼5 kb region downstream of *SDMYB* distal to the structural variant, corresponding to the SD-locus boundaries and region of trans-species polymorphism. For the coding sequence of *SDMYB*, we performed a codon-aware alignment and truncated the alignment after the frame shift in the final exon. For the other three non-coding regions, we used blastn to identify and extract homologous regions from each assembly, reverse complementing as needed, then used muscle (94) to align these regions. Alignments were trimmed with trimal (95), and further curated to remove regions where homology could not be confidently determined. We then fit an edge-proportional partition model to the alignments in IQTree (89), specifying a MG+F1X4+G4 codon substitution model (96) for the coding sequence of *SDMYB* and the HKY+F+G4 nucleotide substitution model (97) for the non-coding regions. We rooted the tree with *Sextonia rubra* as an outgroup. We time-calibrated the maximum likelihood tree assuming a strict clock in the chronos function implemented in the R package ape (98). We used the divergence of Perseeae from Cinnamomeae and Laureae as a fixed calibration point, using the mean and 95% HPD lower and upper bounds from our species phylogeny to give generate a CI for the divergence time of the SD-locus polymorphism.

### Gene Expression Sampling Design and Analysis.

We studied temporal patterns of gene expression in relation to floral anthesis and sex expression in *P. americana*, *P. caerulea*, and *M. thunbergii*, representing three divergent clades within Perseeae (Fig. 3*A*). In *P. americana*, we sampled three biological replicates (not clones) at regular three hour intervals over a 21 h period on April 11th, 2024, from the same plot at UC Lindcove Research and Extension Center in Exeter, CA. Individual flowers were marked during first anthesis on the previous day using jeweler tags. Sampling began at 4:30 AM and continued until 1:30 AM the following morning. Individual flowers were removed at the receptacle and immediately frozen in liquid nitrogen.

Samples for the expression time series of *P. caerulea* and *P. palustris* were sampled from Field 43 at UC South Coast Research and Extension Center in Irvine, CA on June 21, 2024. In this case, we sampled three time points during each of the two flowering phases in a single day. One of the A-type individuals sampled was the same individual used for genome assembly. Samples for the expression time series of *M. thunbergii* were taken from a single individual with A-type flowering on May 2, 2025, at Sonoma Botanical Garden, Glen Ellen, CA.

RNA was extracted from whole flowers using the Qiagen RNAeasy Plant kit. Library prep and Illumina sequencing was performed by Signios Bio, Inc., Foster City, CA. We quantified transcript counts using salmon (99). Raw RNAseq reads were trimmed using skewer (100), and aligned to the Hass transcriptome (40). To quantify both total and allele-specific expression of the three initial candidate genes through time, we used a competitive mapping approach by including both dominant and recessive alleles in the reference transcriptome. For *P. caerulea*, *P. palustris* and *M. thunbergii* we used liftoff (101) to port the Hass annotation to these assemblies and extract the transcriptome.

We normalized transcript counts using the model of DESeq2 (102). We defined a set of 20,096 flower-expressed genes as those with a transcript count greater than or equal to 6 in all samples for at least 3 time points. We used PCA to examine the major axes of floral gene expression variation across all samples. Here, we used redundancy analysis to first remove axes of variation associated with individual replicates using a design matrix to specify individual of origin (this had little effect on the first two axes of unconstrained variation). PCA was run for all samples combined (Fig. 2*A*), and separately for only open flower samples (Fig. 2*B*). To test for rhythmic gene expression of all flower-expressed genes including *SDMYB*, we used the R package RAIN (103) with time points rounded to the nearest hour. To test for rhythmicity of a given gene in both A- and B-types, we applied the test separately to A- and B-types, and applied the Benjamini–Hochberg procedure to the maximum P-value for each gene (a partial conjunction P-value) to control the false discovery rate at 0.05. To test for a morph difference in overall expression levels of *SDMYB*, we fit a mixed effects model in the R package lmer, specifying sampling time point (as in Fig. 2) and individual as a random effect. We used a similar procedure to test for a difference in the average expression of the two alleles in heterozygotes. We fit a cosinor regression model to the *SDMYB* alleles in avocado using the R package CircaCompare (104) to estimate the phase shift between alleles and test for statistical significance. This method was also used to test for a difference in rhythmicity parameters between the *A2* haplotype in heterozygotes and the haploid equivalent in B-type homozygotes. To test for differences at individual timepoints, we used a likelihood ratio test, where a negative binomial model of transcript counts with flowering type as a predictor was compared to a null model fitted with only an intercept.

### Detection of SDMYB Polymorphism Across Species.

To examine the segregation of the SD-locus haplotypes across Perseeae, we genotyped samples at diagnostic *SDMYB* exonic SNPs in public resequencing data from published studies, including low coverage data (∼2×) (41, 42, 86, 105–109). We first defined exonic, biallelic, trans-specific SNP sites within *SDMYB*. We identified two in the second exon (one is the nonsynonymous substitution in the R2R3 domain) and two in the third exon. The SNPs in each exon are 47 and 15 bp apart, typically spanned by single reads, whereas these sets of SNPs are 6.5 kb apart and should not be spanned by correctly aligned read pairs in these data. We selected one of these SNPs per exon, and obtained allelic depth at these SNPs from alignment files. We calculated genotype likelihoods using combined allele counts at the two SNPs with a binomial probability model allowing for a $1\;\times \;10^{-3}$ probability of sequencing error. Using an equal prior probability for each of the three genotypes, we then computed genotype posterior probabilities (*SI Appendix*, Table S7). If a sample showed zero or only one read covering the chosen SNP site in either exon, we used read counts from the neighboring ascertained diagnostic site within the same exon if the depth was higher at that site. The frame-shift mutation in the final exon is also conserved across Perseeae, and we noted that samples with high posterior probability of being heterozygote based on these SNPs also showed the frame shift polymorphism with sufficient depth (*SI Appendix*, Fig. S5). For any individuals polymorphic for the complex frame-shift mutation, which we considered highly unlikely to result from sequencing error (mutation from one allele to the other requires at least 2 insertion/deletion mutations), we set the genotype posterior to be 1 for the heterozygote. To visually demonstrate that the segregation of TSP SNPs in individuals in additional species of unknown phenotype was restricted to *SDMYB*, we visualized SNP sharing with *P. americana* Hass in exonic regions for a genomic window surrounding *SDMYB* with multiple genes on either side (*SI Appendix*, Fig. S5).

## Supplementary Material

Appendix 01 (PDF)

Dataset S01 (XLSX)

## Data Availability

Genome assemblies of *Persea americana* Puebla, *Machilus thunbergii, P. caerulea*, and *P. podadenia* are available at NCBI through BioProjects PRJNA1392525, PRJNA1392526, PRJNA1392542, PRJNA1392543, PRJNA1392566, PRJNA1392567, PRJNA1392587, and PRJNA1392588, respectively (110). Whole genome sequencing data and RNA-seq data are available through NCBI BioProjects PRJNA1393340 and PRJNA1397474 (110). Movies S1–S7 are available at https://zenodo.org/records/18021169 (111). Sequenced individuals of *P. caerulea* and *P. palustris* are deposited at the University of California, Davis herbarium under accession numbers DAV251646 (112), DAV251647 (113), and DAV244839 (114).
