# Supplementary material for: Balanced polymorphism in a floral transcription factor underlies the ancient rhythm of daily sex alternation in avocado
Source: Proc Natl Acad Sci U S A. 2026 Jul 28;123(31):e2606876123. doi: 10.1073/pnas.2606876123 (PMC13438604; doi:10.1073/pnas.2606876123)
Supplement: Supplementary file 1 [file pnas.2606876123.sapp.pdf]

Supporting Information for

Balanced polymorphism in a floral transcription factor underlies the ancient rhythm of daily sex alternation in avocado

Jeffrey S. Groh, Marllon F. Soares dos Santos, Emmanuel Avila de Dios, Gracie Ackerman, Edwin Solares, Rodrigo A. Iturrieta, Eric Focht, Danelle Seymour, Brandon S. Gaut, Mary Lu Arpaia, Graham Coop

Corresponding author: J.S.G.
E-mail: jgroh@berkeley.edu

This PDF file includes:

Supporting text
Figs. S1 to S7
SI References

Supporting Information Text

Extended Methods.

Phenotyping of *Umbellularia californica* We examined flowering phenology of a natural population of California bay laurel *Umbellularia californica* at UC Quail Ridge Reserve in February 2025. Preliminary observations over the span of a day indicated that flowers of this species were not opening and closing within the span of a day. We then tagged up to 10 individual flowers on 17 individuals using jeweler tags and recorded the status of flowers over 5 separate visits in February. Flowers were considered in the female phase if the petals were at least partially open and the stigma appeared mature. Stigmas that appeared moist and whitish/translucent in color were judged as mature, and when they turned brown in color they were scored as finished. The male phase was considered as the period were at least one anther had dehisced, or all anthers had recently dehisced and pollen was still present.

Species phylogeny inference and time calibration Relationships within the ca. 400 species of Perseeae remain unresolved, and previous molecular phylogenetic studies have typically relied on single genes (1–4). Chen et al. 2020 (5) constructed a phylogeny of Lauraceae using 275 orthologs, but with limited taxon sampling from Perseeae. Furthermore, divergence time estimates for Perseeae have rested on either biogeographical assumptions (6), or calibration using a single leaf fossil with uncertain taxonomic placement (2, 7), which may be problematic given the extent of homoplasy in Lauraceae leaf characters (8). To improve phylogenetic resolution in this group, we inferred a phylogeny for 32 Lauraceae taxa (Table S4) using 7,842 single-copy orthologs, and grafting this into a magnoliid backbone tree, we used an alignment of 93 gene trees together with a set of fossil calibrations (Table S5) to time calibrate the tree (Table S6).

First, we identified a set of 7,862 single copy orthologs using fifteen genome assemblies spanning the core Lauraceae (tribes Perseeae, Laureae, and Cinnamomeae) (using OrthoFinder 2.5.5 (9) with option -d to run on DNA sequences). The input transcriptomes consisted of eight de novo genome annotations and liftoff annotations (10) from the avocado genome for unannotated assemblies. After initial ortholog detection, we then used shotgun resequencing data aligned to the *P. americana* genome to generate consensus sequences over these orthologs for an additional sixteen species. To do this, we first generated an all-sites vcf, then used bcftools consensus (11), with parameters -e 'FMT/GT=="./."' -a "N" -M "N" –mark-del "N" for each exonic region of each single copy ortholog, reverse complementing the result for sequences encoded on the reverse strand. Importantly, by treating sites with missing data as 'N' rather than assuming the reference allele (avocado), this approach avoids reference bias from impacting the phylogenetic placement of these samples. We converted any sites called as heterozygous within samples to missing data to avoid any impact of genotype priors and sequencing error in the variant calling step. Doing so, we recovered sequence from all species for 7,842 single copy orthologs, which were then used for phylogenetic inference.

Sequences for each orthogroup were aligned using muscle (12) and subsequently trimmed using trimal using the heuristic algorithm optimized for maximum likelihood phylogeny inference (13). We then inferred a gene tree for each orthogroup using maximum likelihood in iqtree with a GTR+I+G4 substitution model and 1000 ultrafast bootstrap replicates (14). Gene tree nodes with less than 10% bootstrap support were collapsed to polytomies (15) using newick tools https://github.com/xflouris/newick-tools, and then we ran ASTRAL IV (15) on the resulting gene trees to infer a species tree under a model that accounts for incomplete lineage sorting. In parallel, we used a concatenation+ maximum likelihood approach in IQTree using a GTR+I+R4 model with 1000 ultrafast bootstrap replicates. Notably, we recovered identical topologies, with all nodes showing LPP support values of 1 and 100% bootstrap support from these two methods, respectively. We rooted the core-Lauraceae trees at the edge separating Perseeae from Laureae-Cinnamomeae, as the relationships among tribes is well-established, and this was also confirmed in our magnoliid-wide analysis described below.

In parallel we inferred a backbone tree for magnoliids, in order to calibrate our core-Lauraceae tree using established calibration points across magnoliids. First, we used fourteen genome assemblies with independent annotations of protein-coding genes to define a set of 105 single copy orthologs across magnoliids, using OrthoFinder 2.5.5 (9) with coding sequences as input. These orthologs were roughly evenly distributed across all twelve autosomes of avocado. For eleven other genomes for which we could not access annotations, we used liftoff (10) to extract coding sequences of these orthologs, using a reference genome from within the same phylogenetic order and setting parameters '-mm2_options="-r 2k -z 5000 –end-bonus 5" -d 5 -flank 0.2 -polish' to improve alignments between distantly related species, resulting in sequences from at least 95 single copy orthologs for all taxa in our data set. This taxonomic sample spans all four orders within magnoliids and nine families. As described above, we inferred gene trees from trimmed alignments of each ortholog, and inferred species phylogenies using both ASTRAL and concatenation approaches. Importantly, the relationships among broader groups were in perfect agreement with maximal support using both methods, with the exception of relationships within core-Lauraceae, which we confidently resolved in the previous analysis. The tree was rooted along the edge separating the Canellales-Piperales clade from the Magnoliales-Laurales clade following ref. (16).

We time calibrated the tree using the likelihood approximation method of MCMCTree (17). MCMCTree infers node ages using a fixed topology, which is appropriate as both our ASTRAL and concatenation+maximum likelihood analyses gave identical topologies. We specified priors on internal nodes with soft bounds to account for the possibility of fossil misplacement on the phylogeny. Due to the difficulty of unambiguously assigning fossils within Perseeae, we rely on a larger set of fossil calibrations previously established for magnoliids (18), together with additional calibrations for Lauraceae, which are detailed along with justification in Table S5. Briefly, our calibrations incorporate the wide uncertainty in the crown age of magnoliids (19, 20), include established lower bounds for deeper nodes within magnoliids (21), and set priors on ages of nodes within Lauraceae that are consistent with patterns of diversification of this group in the late Cretaceous and Cenozoic (22–25) and

that are consistent with estimates from previous studies (2, 3, 25).

For nodes without specified priors, the priors are defined based on the birth-death model with the default parameters set by the program, which gives a uniform distribution of node ages for uncalibrated nodes, subject to constraints of the use-calibrated nodes. Ninety-three of the 105 single-copy orthologs from our magnoliid-wide orthology search were also contained in the set of single-copy orthologs defined for core Lauraceae. We therefore concatenated the alignments of these genes for the molecular data input to MCMCTree. We ran MCMCTree using the HKY85+G5 substitution model. We specified the correlated rates relaxed clock model (rather than independent rates) as our taxon sampling was highly biased, with many taxa from Lauraceae and few from other major clades within magnoliids. We specified a diffuse prior for substitution rates with the gamma rate parameter $\alpha = 2$ as recommended by the program manual. To set the mean of the prior on substitution rates, we used the mean too to tip height from the maximum likelihood tree of magnoliids together with the mean of the prior on the root age to derive an estimate of substitutions per site per 100 million years. We first ran MCMC algorithm by sampling from the prior to verify that our prior specification was sensible and that marginal priors of fossil-calibrated nodes were not far from the user specification. We ran two separate chains of the MCMC algorithm with substitution data for a sample size of 50,000, sampling every 10 iterations after a burn-in period of 5,000 iterations. Results were checked for convergence across multiple chains.

SD-locus phylogeny. Our genome assembly from an A-type *P. caerulea* individual revealed that it was a homozygote for the *A1* haplotype, thus we were lacking an *A2* sequence for *P. caerulea*. Therefore, we supplemented our genome sequences by extracting exonic *SDMYB* sequence from a B-type individual of *P. caerulea* for which we had performed RNAseq. To do this, we aligned the transcripts to avocado genome using STAR (26) and then used bcftools (11) to extract a consensus sequence from over the exons separately for each sample. Coverage was very high over the exons for the samples used, and we first filtered variants for GQ 40, then combined these with invariant sites. Any sites with missing data were coded as 'N' rather than assuming the reference sequence. We also aligned to the dominant allele of avocado, whereas this B-type sample has two recessive alleles. Thus, reference bias does not impact the phylogenetic placement of these samples. We used a similar procedure to incorporate *Cinnamomum verum* into the phylogeny by extracting a consensus sequence from WGS alignments over the *SDMYB* coordinates in the avocado genome.

Gene expression analyses. For our avocado time series, at each sampling time point, trees were sampled in a consistent order, which alternated between A- and B-types. The total time to sample all 6 trees was approximately 10-15 minutes. Flowers that opened during first anthesis on the day of sampling were also tagged such that they could be sampled after closing. Sampling stopped when flowers had closed after their second opening with the exception of two flowers of B-types. We included an additional time point at the 2nd anthesis of B-types as our regular 3 hour intervals would have otherwise missed this developmental landmark. During the main sampling day and for two nights previous, we used WingScapes BirdCam Pro cameras to photograph inflorescences every 10 minutes and generate time lapse videos of flowering (see Supplementary Videos).

For *Persea caerulea*, one of the A-type individuals sampled was the same individual used for genome assembly, a dominant allele homozygote, while the other individual was heterozygote. For *Machilus thunbergii*, the sampled individual was the same as the one used for the reference genome. Time-lapse photography (Supplementary Videos) confirmed the flowering types of these individuals.

For avocado, we used competitive mapping to the transcriptome by including both alleles for the three initial candidate genes identified in GWAS. To improve mapping quality for these three genes, we used the RNAseq alignments to manually edit the transcript sequences to include UTR sequence, and also in doing so identified that the 'Hass' annotation for MRB53_031467 was missing two exons, which we included in the reference for alignment.

The allele-specificity of our competitive mapping quantification approach depends on the density of allele-specific SNPs in the reference transcriptome copies. In total in avocado, we found 26 SNPs differing between the *A1* and *A2* haplotypes across the three exons of *SDMYB*, 9 SNPs across the 6 exons of *MRB53_031467*, and 3 SNPs across the 8 exons of *MRB53_031468*. Within the mature transcript sequence of *SDMYB*, the largest distance between two haplotype-specific SNPs is 148 bp, and each member of a read pair is 150 bp. Thus, the vast majority of reads will overlap at least one haplotype-specific SNP and we expect our quantification of allele-specific expression to be accurate. In genes *MRB53_031467* and *MRB53_031468*, the longest portion of the transcripts containing no haplotype-specific SNPs are approximately 670 bp and 950 bp, representing approximately 49% and 54% of the total transcript lengths. The majority of reads are expected to overlap at least one SNP, but those that don't could plausibly align with equal probability to either of the two alleles, making quantification of allele-specific expression conservative. Thus, as a complementary approach, we separately quantified allele-specific expression by aligning all reads to the same reference and directly measuring allelic depth at haplotype-specific SNPs, taking a depth weighted average of relative allele depth across SNPs. We found highly similar results using both approaches and report the results from the competitive mapping.

In avocado, for the three genes where we considered alleles separately, we summed the normalized counts across alleles to obtain a gene-level normalized transcript count.

To measure expression of the duplicate copies of *MRB53_031467* present in the *P. caerulea* dominant haplotype we used RNAseq alignments to extract the coding regions corresponding to both copies. Our *P. caerulea* assembly was from a dominant homozygote individual, so to obtain the coding sequence of *SDMYB* for the recessive haplotype, we used the short read RNAseq data from the B-type individual of *P. caerulea*. Here, we extracted the consensus sequence over the exons and randomly resolved any heterozygous sites to one allele.

Supplementary Table Legends. Supplementary Tables are provided separately in a xlsx file.

Table S1 Schematic of sampling design for time course of gene expression from avocado flowers.

Table S2 Sample information for flowers used for RNAseq.

Table S3 Sample information and quality metrics for de novo genome assemblies.

Table S4 Sources of genomes used to infer the Magnoliid phylogeny shown in Fig. S3.

Table S5 Fossil calibrations used for the Magnoliid phylogeny with brief justifications and references.

Table S6 Divergence time estimates for a set of nodes in our inferred phylogeny.

Table S7 Posterior probabilities of *SDMYB* diploid genotypes for individuals of tribe Perseeae with whole genome sequence data available.

Supplementary Video Legends. Supplementary Videos are available at https://doi.org/10.5281/zenodo.18021169.

Video S1 Time lapse video of *Persea avocado* 'Hass' flowers (A-type).

Video S2 Time lapse video of *Persea avocado* 'Zutano' flowers (B-type).

Video S3 Time lapse video of *Persea avocado* 'Holiday' flowers (A-type).

Video S4 Time lapse video of *Machilus thunbergii* flowers (A-type).

Video S5 Time lapse video of *Persea caerulea* flowers (B-type).

Video S6 Time lapse video of *Persea caerulea* flowers (A-type).

Video S7 Time lapse video of *Persea caerulea* flowers (A-type).

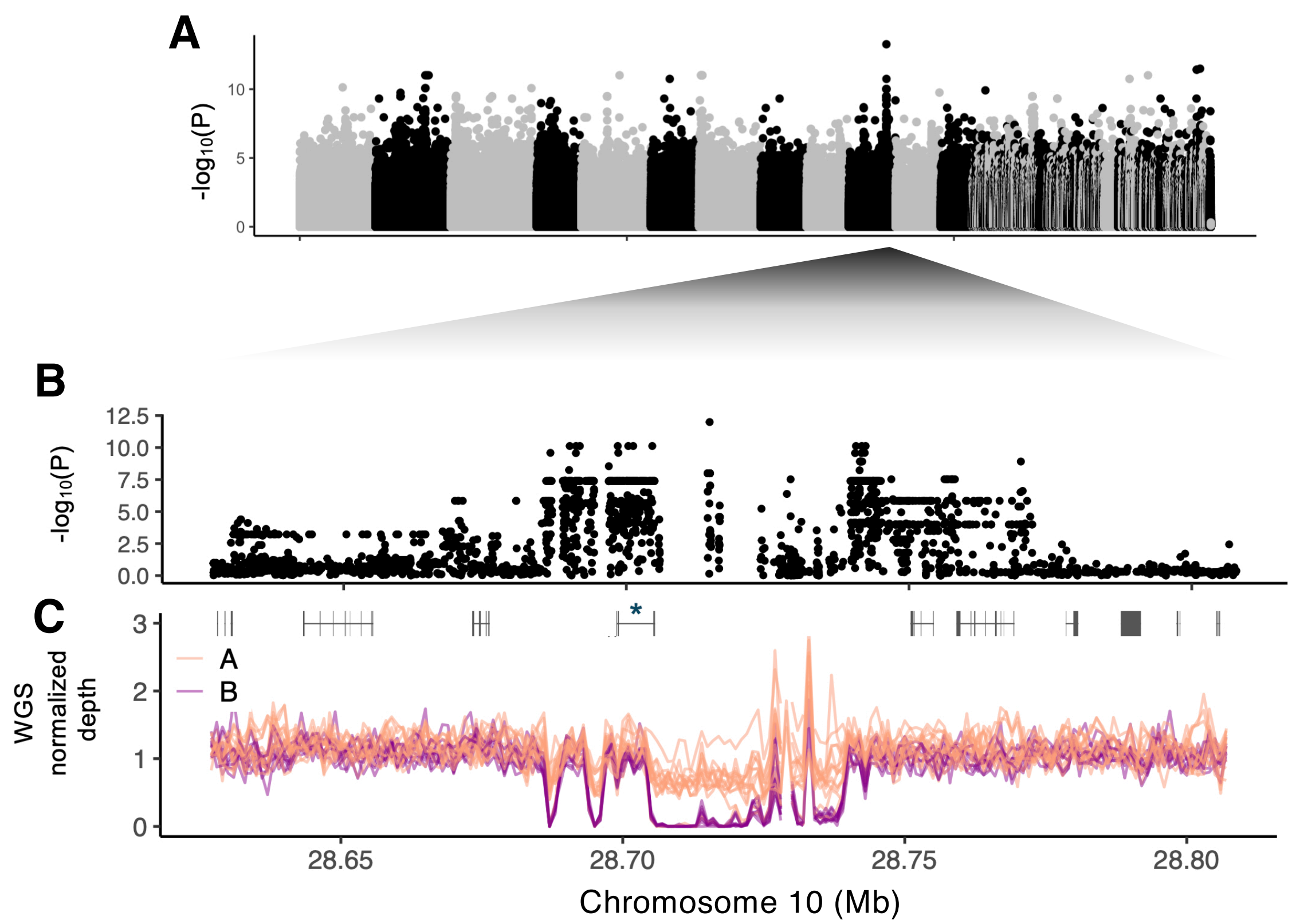

Fig. S1. A) GWAS using published data (27, 28) of 23 avocado varieties not in our mapping population against an assembly of 'Gwen' (27) **B)** Zoomed in view of the GWAS peak region in the 'Hass' alternate assembly (29) with gene models at bottom. **C)** Normalized read depth in 1 kb windows across this region for the same samples.

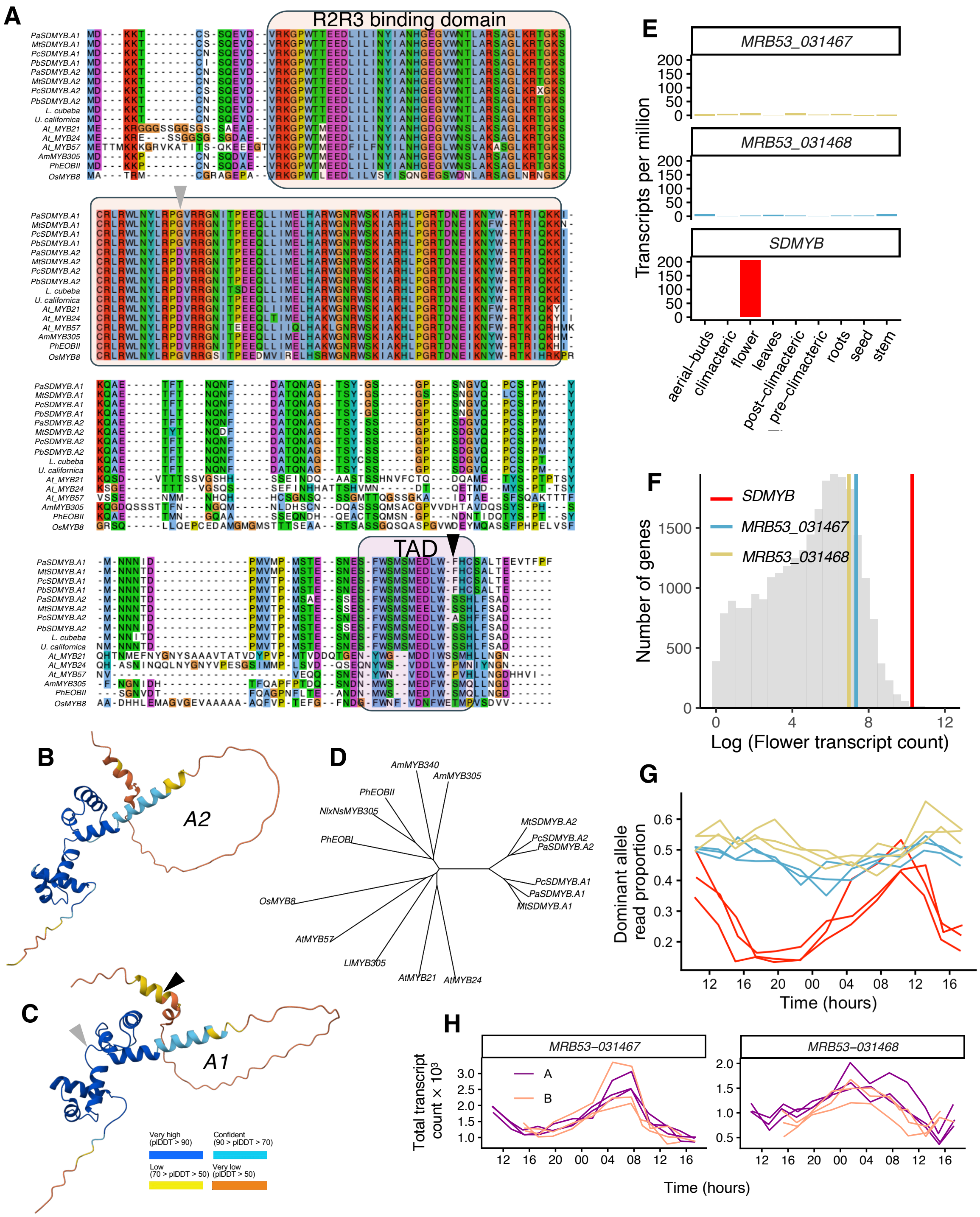

Fig. S2. A) Alignment of SG19 R2R3 MYB amino acid sequences. Species abbreviations as follows: Pa=*Persea americana*, Mt=*Machilus thunbergii*, Pc=*Persea caerulea*, Pb=*Phoebe bournei*, followed by *Litsea cubeba*, *Umbellularia california*, At=*Arabidopsis thaliana*, Am=*Antirrhinum majus*, Ph=*Petunia hybrida*, and Os=*Oryza sativa*. Colored triangles above alignment highlight differences between SDMYB alleles within conserved domains. **B,C)** AlphaFold predictions of protein structure for the A2 (recessive) and A1 (dominant) avocado alleles. The high-confidence structured region (dark blue) corresponds to the R2R3 MYB DNA binding domain. The TAD structure has lower confidence but contains a predicted alpha-helix. Black and gray triangles show positions of derived changes in A1, corresponding to those shown in (A). **D)** Neighbor Joining tree based on a trimmed amino acid alignment of SG19 R2R3 MYB sequences. Species codes as in (A) with the addition of Ll=*Lilium longiflorum*. Note the placement of the root is uncertain. **E)** *SDMYB* shows flower-specific expression, unlike the other two genes near the association peak. Data are from a single avocado individual (30) used elsewhere for genome assembly (28), whose genotype we identified as *A2/A2* from published genomic sequence. **F)** Average expression level of all flower-expressed avocado genes across 73 samples (Table S2). The expression levels of three genes at the SD-locus are noted by colored vertical lines. **G)** Proportion of transcripts derived from the dominant haplotype for three genes at the association peak. Colors are the same as in (F). **H)** Total transcripts (both alleles) by flowering type of the two additional genes besides *SDMYB* at the association peak.

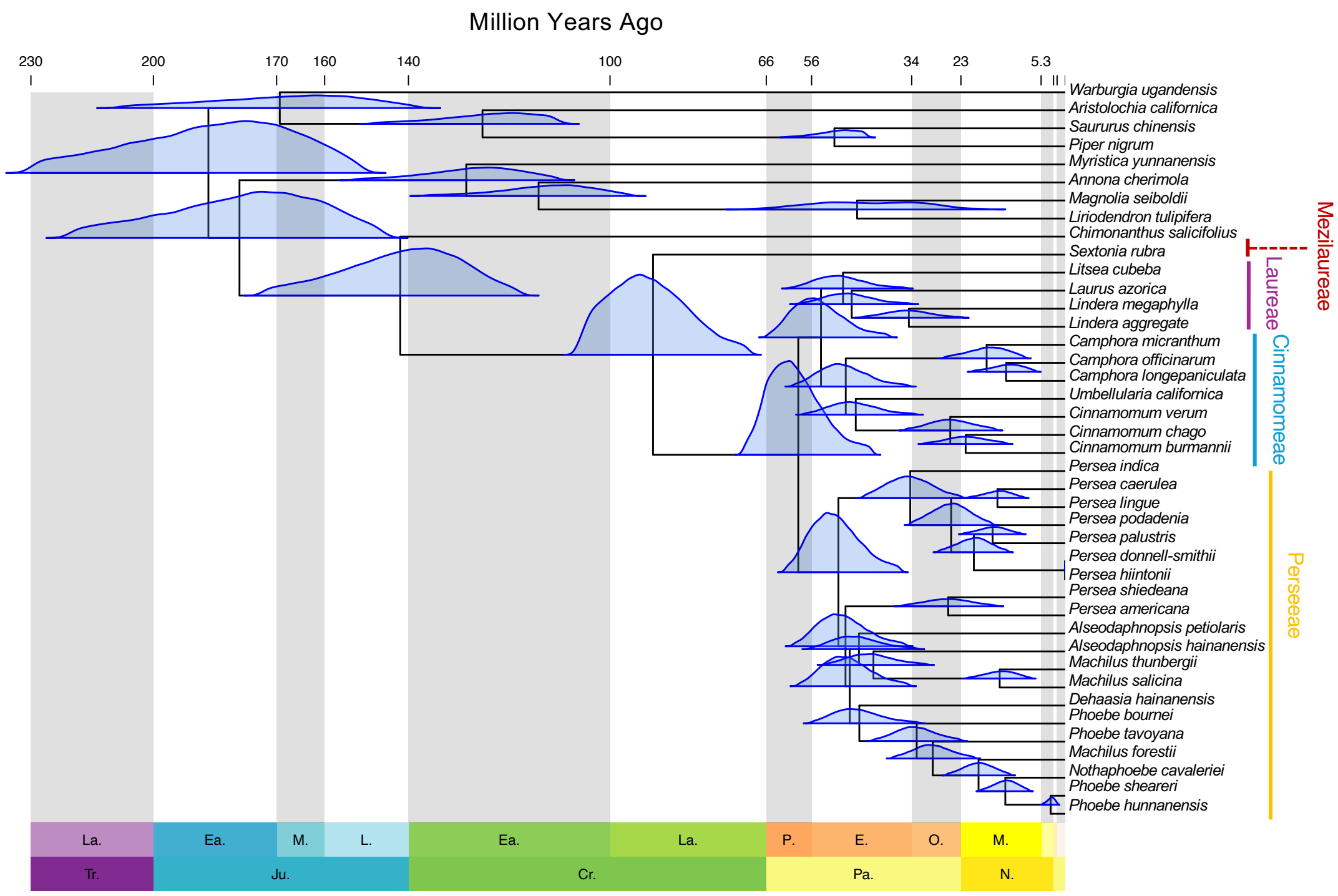

Fig. S3. A) Fossil-calibrated phylogeny of a subset of Magnoliids. Taxon sampling is targeted to Lauraceae and the inclusion of at least one members of each magnoliid order, but is not meant to be exhaustive and reflects availability of public whole genome sequence data suitable for our analysis method. The blue distributions are posterior probability distributions from MCMCTree (see Materials and Methods. Point estimates and credible intervals for several nodes are reported in Table S6. See Table S5 for fossil calibrations. Not all Lauraceae tribes are shown, see (31) for a review of broad level taxonomy in Lauraceae. All nodes in the tree shown had maximum support in both ASTRAL and concatenation + maximum likelihood methods.

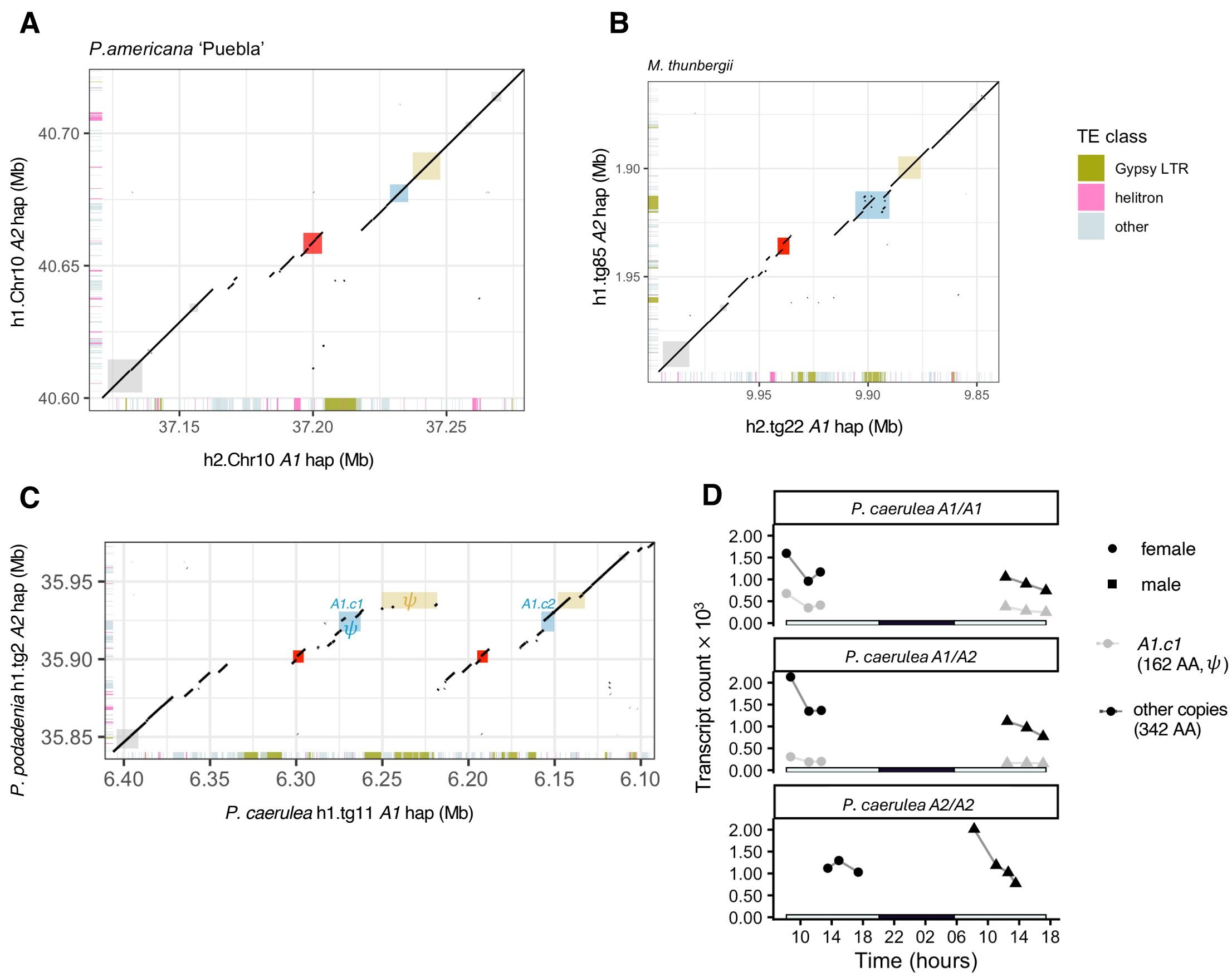

Fig. S4. A-C) Dotplot alignments between alternative SD-locus haplotypes within *P. americana* (A), *M. thunbergii* (B), and *Persea* subg. *Eriodaphne* (C). Syntenic gene regions are highlighted in center with colored coxes, *SDMYB* in red. Transposable element annotations are shown at edges with two classes indicated in color. In (C), ψ denotes predicted pseudogenes in the *A1* haplotype of *P. caerulea*. Two duplicate copies of *MRB53-031467* are labeled in blue, see also panel (D). **D)** Expression of two paralogs of *MRB53-031467*, the gene downstream to *SDMYB* copies within the dominant haplotype of *P. caerulea*. Panels represent different individuals of *P. caerulea*. We were unable to differentiate between the transcripts from the recessive haplotype copy of this gene and the 2nd copy found on the dominant haplotype, *A1.c2*, so transcript counts from these are pooled. However in the *A1/A1* homozygote (top) we can be sure this pool represents only the *A1.c2* copy. In this individual (top), *A1.c1* shows low expression and has a truncated amino acid sequence, appearing to be a pseudogene.

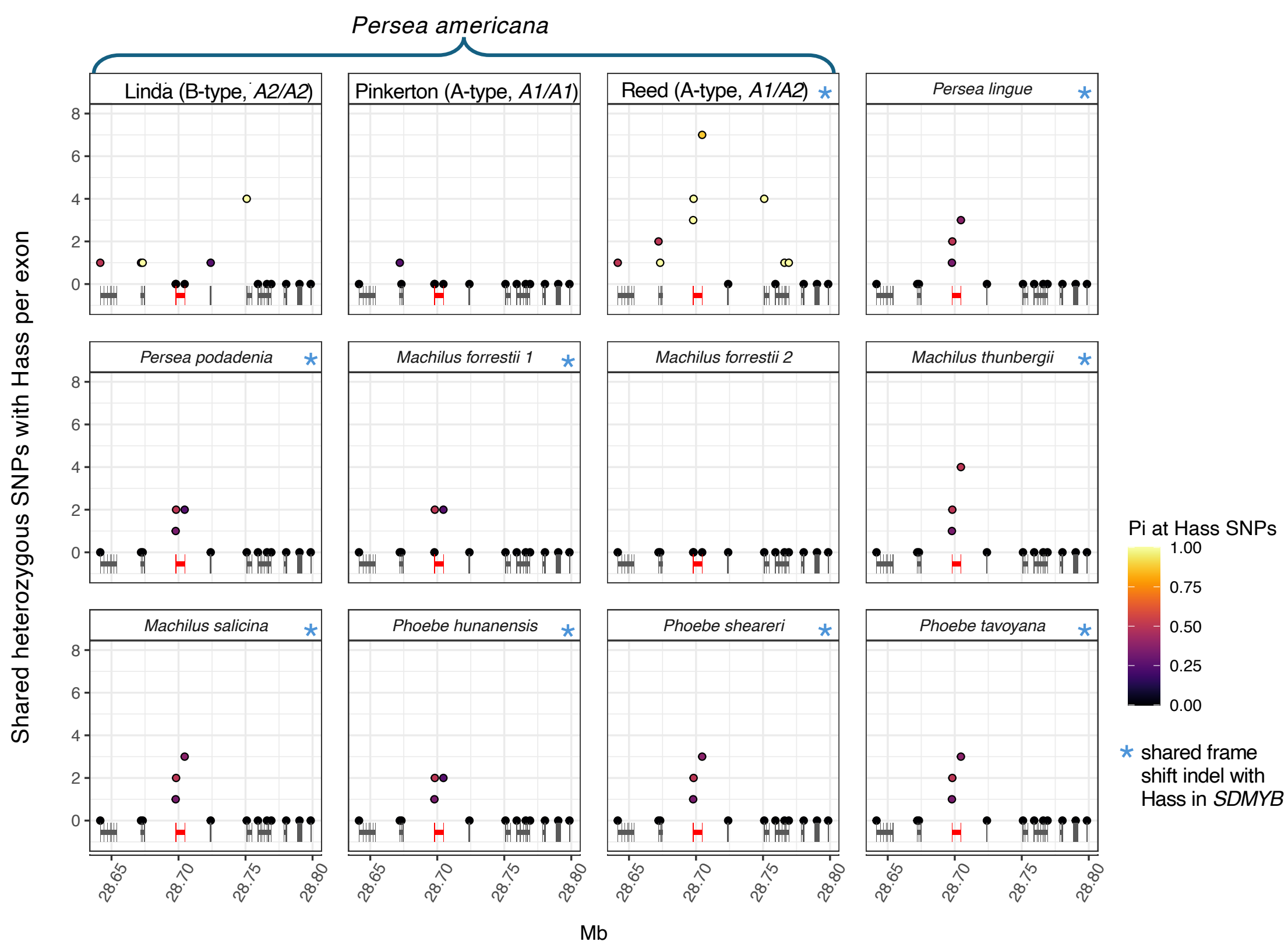

Fig. S5. Shared heterozygous sites with *Persea americana* 'Hass' (A-type, *A1/A2*) in exonic sequence for a subset of the individuals examined (see Table S4 for full results). Each point represents a single exon, with the height of the point representing the number of sites that are shared SNP polymorphisms with 'Hass'. The color of each point represents the fraction of sites the individual is heterozygous at, out of the total number of heterozygous sites in 'Hass' for that exon. In addition, blue asterisk denotes the presence of a shared frame shift polymorphism in the last exon of *SDMYB*, which coincides with the presence of shared SNP polymorphisms.

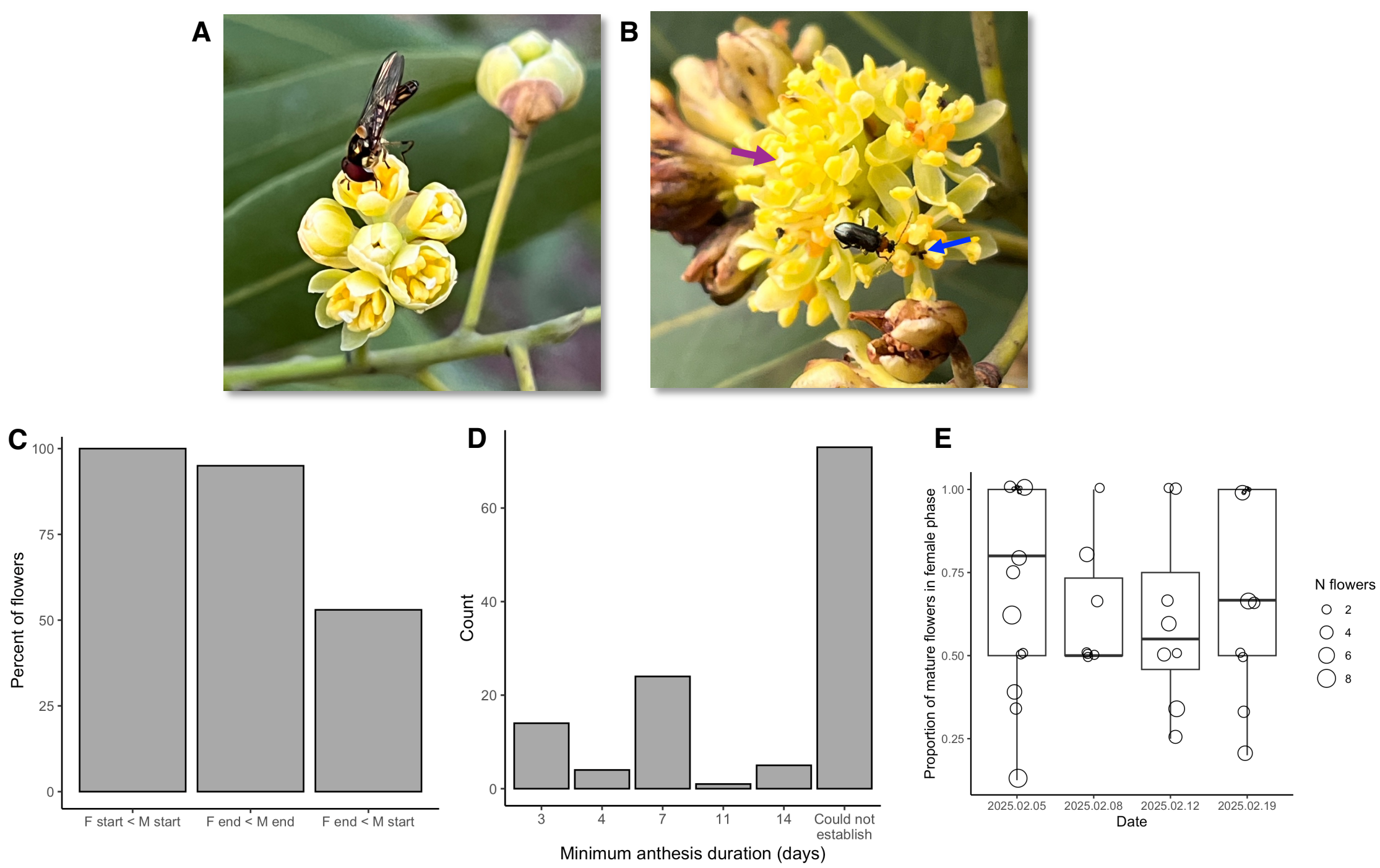

Fig. S6. A) Flowers of *Umbellularia californica* in female phase. Stigmas appear moist and whitish/translucent, while anther valves have not dehisced. **B)** An example of overlap between female and male phase between flowers on the same plant. Purple arrow indicates a flower in female phase with a fresh stigma and undehisced anthers. Blue arrow indicates a flower with a dark whithered stigma and dehisced anthers. **C)** Summary of intrafloral dichogamy. All flowers are protogynous: the female phase always begins before the male phase and almost always ends before the male phase ends, with some degree of overlap based on visual assessment. F and M mark female and male respectively. **D)** A considerable proportion of flowers showed anthesis periods spanning more than several days. The x-axis gives the minimum number of days a tagged flower was inferred to be sexually mature over the multiple field site visits. This minimum couldn't be established if a flower went from unopened to completion of anthesis over two consecutive visits (the majority of cases), or if observations did not span the start and end of floral anthesis. **E)** Dichogamy is not synchronous between flowers on the same plant. Each dot shows a tree measured on a particular date, the size indicating the number of tagged, mature flowers open. See 'Phenotyping' section in Methods for details.

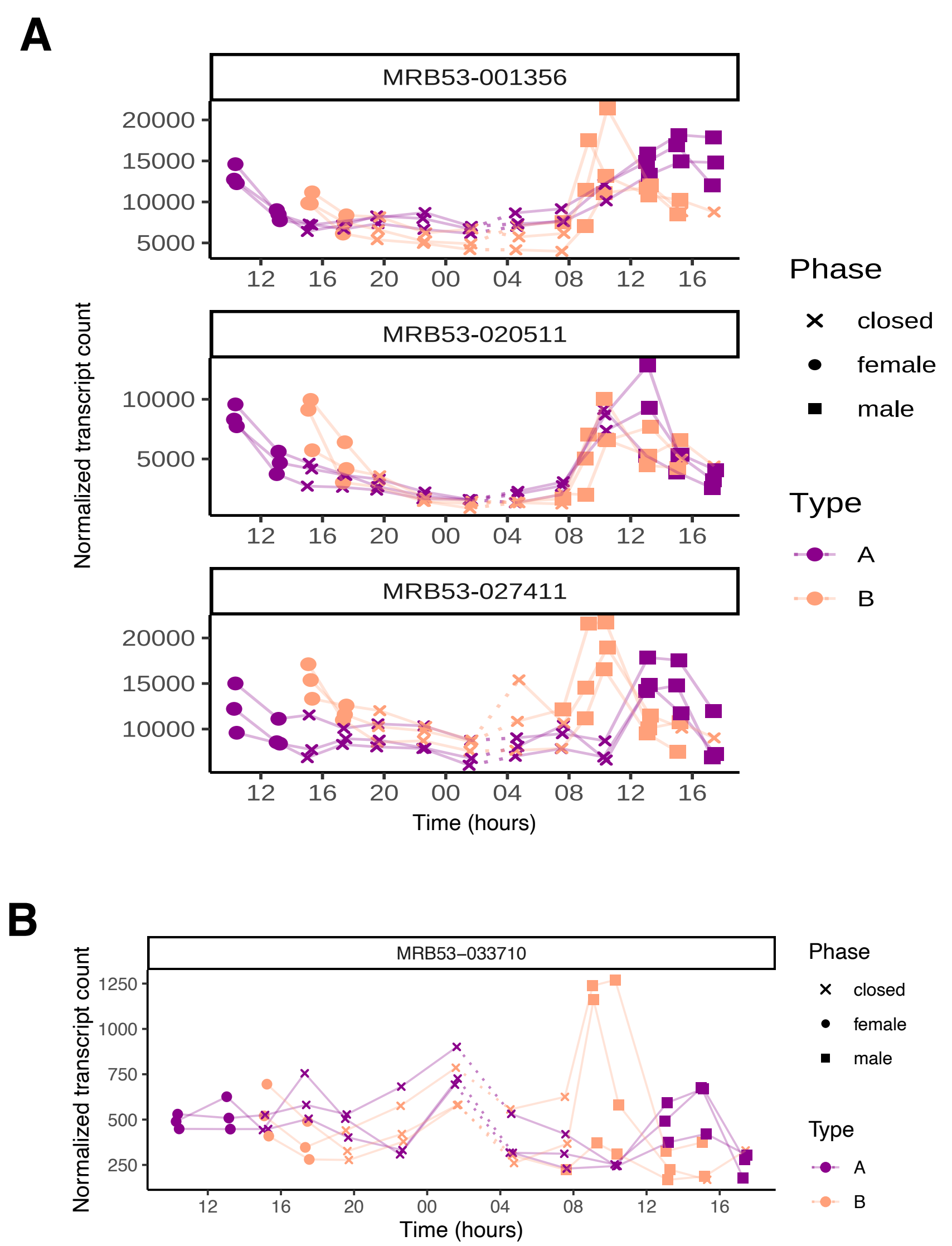

Fig. S7. A) Several avocado homologs of the Arabidopsis *JAZ* genes show morph-specific expression patterns in avocado, suggesting that these are regulated by *SDMYB*. **B)** The avocado ortholog of *Alpinia mutica* Am*SMPED1* shows evidence of morph-specific expression, particularly at second anthesis.

References

1. JG Rohwer, et al., Is *Persea* (Lauraceae) monophyletic? Evidence from nuclear ribosomal ITS sequences. *Taxon* **58**, 1153–1167 (2009).
2. L Li, et al., Molecular phylogenetic analysis of the Persea group (Lauraceae) and its biogeographic implications on the evolution of tropical and subtropical Amphi-Pacific disjunctions. *Am. J. Bot.* **98**, 1520–1536 (2011).
3. TW Xiao, HF Yan, XJ Ge, Plastid phylogenomics of tribe Perseeae (Lauraceae) yields insights into the evolution of East Asian subtropical evergreen broad-leaved forests. *BMC Plant Biol.* **22**, 32 (2022).
4. A Chanderbali, D Soltis, P Soltis, B Wolstenholme, Taxonomy and botany in *The avocado: botany, production and uses*, eds. AW Whiley, B Schaffer, BN Wolstenholme. (CABI GB), pp. 23–44 (2024).
5. YC Chen, et al., The *Litsea* genome and the evolution of the laurel family. *Nat. Commun.* **11**, 1675 (2020).
6. AS Chanderbali, H Van Der Werff, SS Renner, Phylogeny and historical biogeography of Lauraceae: evidence from the chloroplast and nuclear genomes. *Annals Mo. Bot. Gard.* pp. 104–134 (2001).
7. J Li, J Qiu, W Liao, J Jin, Eocene fossil *Alseodaphne* from Hainan Island of China and its paleoclimatic implications. *Sci. China Ser. D: Earth Sci.* **52**, 1537–1542 (2009).
8. MA Rubalcava-Knoth, SRS Cevallos Ferriz, Implications of lack of morphological information in fossil leaves related to Lauraceae: a statistical evaluation. *J. Syst. Palaeontol.* **23**, 2432269 (2025).
9. DM Emms, S Kelly, OrthoFinder: phylogenetic orthology inference for comparative genomics. *Genome Biol.* **20**, 1–14 (2019).
10. A Shumate, SL Salzberg, Liftoff: accurate mapping of gene annotations. *Bioinformatics* **37**, 1639–1643 (2021).
11. P Danecek, et al., Twelve years of SAMtools and BCFtools. *GigaScience* **10**, giab008 (2021).
12. RC Edgar, MUSCLE: multiple sequence alignment with high accuracy and high throughput. *Nucleic Acids Res.* **32**, 1792–1797 (2004).
13. S Capella-Gutiérrez, JM Silla-Martínez, T Gabaldón, trimAl: a tool for automated alignment trimming in large-scale phylogenetic analyses. *Bioinformatics* **25**, 1972–1973 (2009).
14. LT Nguyen, HA Schmidt, A Von Haeseler, BQ Minh, IQ-TREE: a fast and effective stochastic algorithm for estimating maximum-likelihood phylogenies. *Mol. Biol. Evol.* **32**, 268–274 (2015).
15. C Zhang, M Rabiee, E Sayyari, S Mirarab, ASTRAL-III: polynomial time species tree reconstruction from partially resolved gene trees. *BMC Bioinforma.* **19**, 15–30 (2018).
16. AJ Helmstetter, et al., Toward a phylogenomic classification of magnoliids. *Am. J. Bot.* p. e16451 (2025).
17. Z Yang, B Rannala, Bayesian estimation of species divergence times under a molecular clock using multiple fossil calibrations with soft bounds. *Mol. Biol. Evol.* **23**, 212–226 (2006).
18. J Massoni, TL Couvreur, H Sauquet, Five major shifts of diversification through the long evolutionary history of Magnoliidae (angiosperms). *BMC Evol. Biol.* **15**, 1–14 (2015).
19. H Sauquet, S Ramírez-Barahona, S Magallón, What is the age of flowering plants? *J. Exp. Bot.* **73**, 3840–3853 (2022).
20. AR Zuntini, et al., Phylogenomics and the rise of the angiosperms. *Nature* **629**, 843–850 (2024).
21. J Massoni, J Doyle, H Sauquet, , et al., Fossil calibration of magnoliidae, an ancient lineage of angiosperms. *Palaeontol. Electron.* **18.1.2FC**, 1–25 (2015).
22. DW Taylor, Eocene floral evidence of Lauraceae: corroboration of the North American megafossil record. *Am. J. Bot.* **75**, 948–957 (1988).
23. JA Doyle, PK Endress, Integrating Early Cretaceous fossils into the phylogeny of living angiosperms: Magnoliidae and eudicots. *J. Syst. Evol.* **48**, 1–35 (2010).
24. EM Friis, PR Crane, KR Pedersen, *Early flowers and angiosperm evolution.* (Cambridge University Press), (2011).
25. JF Huang, et al., Origins and evolution of cinnamon and camphor: A phylogenetic and historical biogeographical analysis of the *Cinnamomum* group (Lauraceae). *Mol. Phylogenetics Evol.* **96**, 33–44 (2016).
26. A Dobin, et al., STAR: ultrafast universal RNA-seq aligner. *Bioinformatics* **29**, 15–21 (2013).
27. E Solares, et al., Insights into the domestication of avocado and potential genetic contributors to heterodichogamy. *G3: Genes, Genomes, Genet.* **13**, jkac323 (2023).
28. M Rendón-Anaya, et al., The avocado genome informs deep angiosperm phylogeny, highlights introgressive hybridization, and reveals pathogen-influenced gene space adaptation. *Proc. Natl. Acad. Sci.* **116**, 17081–17089 (2019).
29. O Nath, et al., A haplotype resolved chromosomal level avocado genome allows analysis of novel avocado genes. *Hortic. Res.* **9**, uhac157 (2022).
30. E Ibarra-Laclette, et al., Deep sequencing of the Mexican avocado transcriptome, an ancient angiosperm with a high content of fatty acids. *BMC genomics* **16**, 1–18 (2015).
31. L Li, et al., Global advances in phylogeny, taxonomy and biogeography of Lauraceae. *Plant Divers.* **47**, 341–364 (2025).
